# Thermal Processing Creates Water‐Stable PEDOT:PSS Films for Bioelectronics

**DOI:** 10.1002/adma.202415827

**Published:** 2025-03-03

**Authors:** Siddharth Doshi, Margaux O. A. Forner, Pingyu Wang, Salim El Hadwe, Amy T. Jin, Gerwin Dijk, Kenneth Brinson, Juhwan Lim, Antonio Dominguez‐Alfaro, Carina Yi Jing Lim, Alberto Salleo, Damiano G. Barone, Guosong Hong, Mark L. Brongersma, Nicholas A. Melosh, George G. Malliaras, Scott T. Keene

**Affiliations:** ^1^ Department of Materials Science and Engineering Stanford University Stanford CA 94305 USA; ^2^ Electrical Engineering Division Department of Engineering University of Cambridge Cambridge CB3 0FA UK; ^3^ Department of Clinical Neurosciences University of Cambridge Cambridge Biomedical Campus Cambridge CB2 0QQ UK; ^4^ Cavendish Laboratory Department of Physics University of Cambridge Cambridge CB3 0HE UK; ^5^ Department of Materials Science and NanoEngineering Rice University Houston TX 77005 USA

**Keywords:** conductive polymers, electrochemical transistors, implantable probes, laser patterning, polymer processing

## Abstract

Organic mixed ionic‐electronic conductors have emerged as a key material for the development of bioelectronic devices due to their soft mechanical properties, biocompatibility, and high volumetric capacitance. In particular, PEDOT:PSS has become a choice material because it is highly conductive, easily processible, and commercially available. However, PEDOT:PSS is dispersible in water, leading to delamination of films when exposed to biological environments. For this reason, chemical cross–linking agents such as (3‐glycidyloxypropyl)trimethoxysilane (GOPS) are used to stabilize PEDOT:PSS films in water, but at the cost of decreased electrical performance. Here, it is shown that PEDOT:PSS thin films become water‐stable by simply baking at high temperatures (>150 °C) for a short time (≈ 2 min). It is shown that heat‐treated PEDOT:PSS films are as stable as their chemically‐cross–linked counterparts, with their performance maintained for >20 days both in vitro and in vivo. The heat‐treated films eliminate electrically insulating cross–linkers, resulting in a 3× increase in volumetric capacitance. Applying thermal energy using a focused femtosecond laser enables direct patterning of 3D PEDOT:PSS microstructures. The thermal treatment method is compatible with a wide range of substrates and is readily substituted into existing workflows for manufacturing devices, enabling its rapid adoption in the field of bioelectronics.

## Graphical abstract

Instead of using chemical cross–linkers, it is shown that PEDOT:PSS thin films for bioelectronics become water‐stable after a simple heat treatment. The heat treatment is compatible with a range of rigid and elastomeric substrates and films are stable in vivo for >20 days. 2D and 3D PEDOT:PSS structures can be patterned by delivering heat with a focused femtosecond laser.

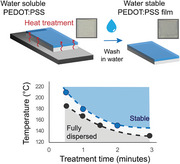

## 1 Introduction

Poly(3,4‐ethylenedioxythiophene) polystrene sulfonate (PEDOT:PSS) is a widely used semiconducting polymer that has emerged as a promising materials candidate for a diverse array of technological applications, ranging from organic electrochemical transistors (OECTs),^[^ ^1^, ^2^, ^3^ ^]^ bioelectronic devices,^[^ ^4^, ^5^, ^6^ ^]^ energy storage devices,^[^ ^7^ ^]^ solar cells,^[^ ^8^ ^]^ nanophotonic devices,^[^ ^9^, ^10^ ^]^ and electrochromic displays.^[^ ^11^, ^12^ ^]^ The unique behavior of PEDOT:PSS comes from its ability to couple ionic and electronic charges by applying a potential against an electrolyte, intercalating ions from electrolyte solution into the bulk of the material, resulting in a change in electronic and optical properties.^[^ ^13^ ^]^

In transistors, this property allows the source‐to‐drain current to be electrochemically modulated by applying a bias to the gate electrode, enabling devices ranging from biosensors^[^ ^14^, ^15^, ^16^ ^]^ to neuromorphic electronics.^[^ ^17^, ^18^ ^]^ Successfully leveraging these unique properties for functional devices commonly requires that PEDOT:PSS structures be operated in aqueous electrolytes. However, commercially available PEDOT:PSS is dispersible in water.^[^ ^19^ ^]^ As a result, thin films of PEDOT:PSS delaminate when submerged in water, presenting a key challenge limiting the lifetime and performance of these devices with aqueous electrolytes. While there are alternative mixed conducting polymers that do not dissolve in aqueous environments such as poly(2‐(3,3′‐bis(2‐(2‐(2‐methoxyethoxy)ethoxy)ethoxy)‐[2‐2′‐bithiophen]‐5‐yl)thieno[3,2‐b]thiophene) (p(g2T‐TT))^[^ ^20^ ^]^ and poly(benzimidazobenzophenanthroline) (BBL),^[^ ^21^ ^]^ PEDOT:PSS‐based materials have high electronic mobilities,^[^ ^22^ ^]^ have been used in humans,^[^ ^23^ ^]^ and PEDOT:PSS is synthesized at a much larger scales than alternative options. Therefore, it is critical to improve the stability of PEDOT:PSS thin films to make progress toward translation of conjugated polymer‐based bioelectronics.

Typically, chemical additives are utilized to improve the adhesion of PEDOT:PSS thin films and prevent their dissolution in water. However, these often affect the performance and stability of the resultant devices. The most widely used treatment involves using (3‐glycidyloxypropyl)trimethoxysilane (GOPS) as cross–linking agent which is directly added into the PEDOT:PSS dispersion. Unfortunately, addition of GOPS can reduce the electronic and ionic conductivity of PEDOT:PSS thin films by over an order of magnitude.^[^ ^19^ ^]^ The transport properties of the films are also highly sensitive to the GOPS concentration,^[^ ^24^ ^]^ leading to variability in batch‐to‐batch device performance. Many other reported chemical treatment processes are often substrate or formulation‐specific, limiting process flexibility and constraining the design of functional organic electronic devices. Recently reported approaches to induce water stability involve exposure of the material to photothermal treatments and electron‐beam radiation. These approaches involve direct exposure of the PEDOT:PSS films to laser^[^ ^25^, ^26^ ^]^ and electron‐beam irradiation.^[^ ^9^ ^]^ There is also recent work showing that functionalizing the substrate surface with GOPS is sufficient to keep PEDOT:PSS adhered to the substrate while avoiding cross–linking of the entire film, thereby improving device performance.^[^ ^27^ ^]^ These promising approaches demonstrate that the structure and properties of PEDOT:PSS films can be modified by the application of photothermal and kinetic energy, suggesting the possibility of non‐chemical routes to realizing stable semiconducting devices with coupled ionic‐electronic conduction.

**Figure 1:**
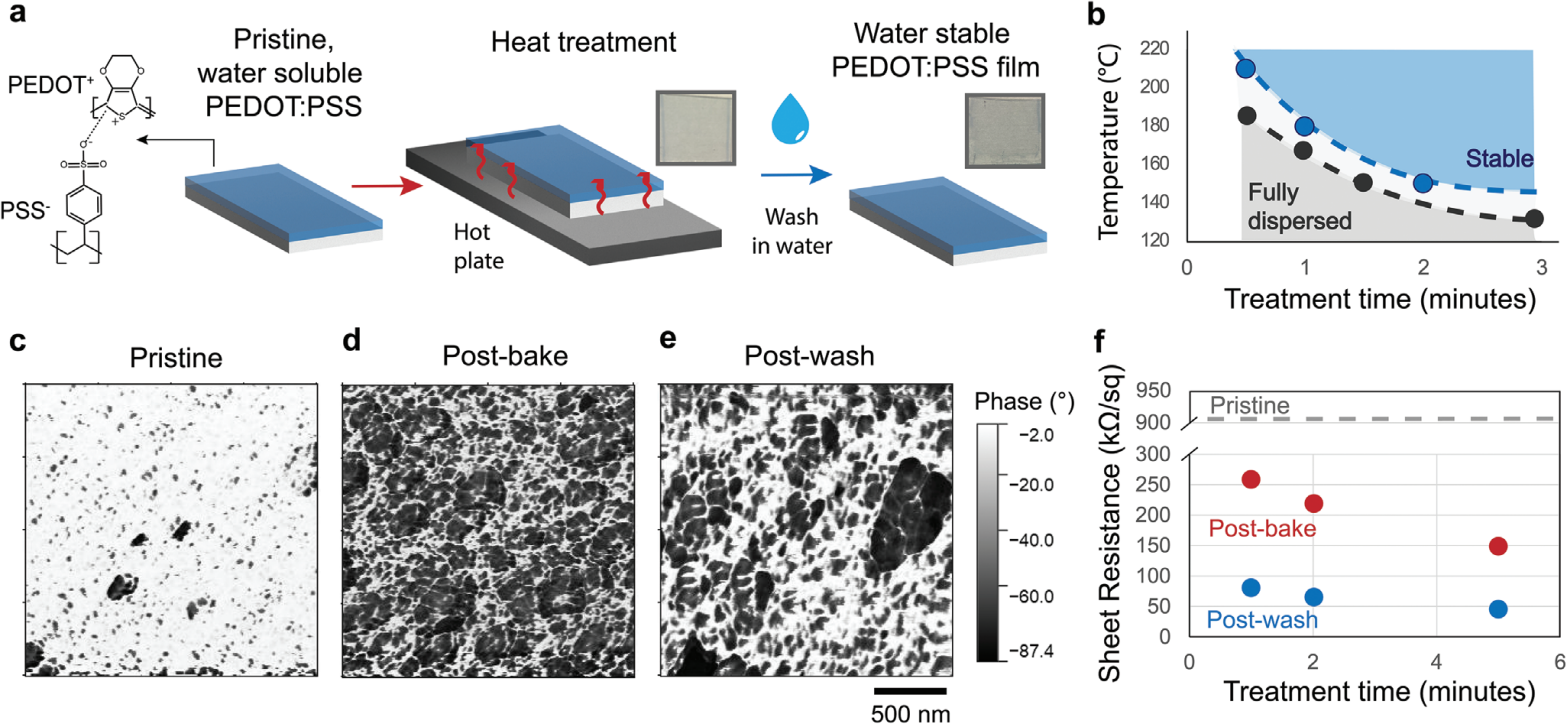
Heat treatment yields water‐stable PEDOT:PSS thin films. a) Schematic showing the heat treatment process, which involves a simple thermal bake on a standard hot plate. Films are no longer dissolved upon exposure to water. b) Landscape of parameters yielding films that remain stable in water (green) and those that are partially dissolved (grey) and fully dissolved (orange) upon exposure to water. c) AFM phase measurements for PEDOT:PSS films in the pristine state prior to treatment, d) after heat treatment at 180 °C for 2 mins, and e) after exposure to water. (c–e) Share a scale bar and color bar. f) Effect of treatment time on the sheet resistance of films baked at 180 °C, measured with a four‐point probe test, both prior to and after exposure to water.

Here, we find that a simple high‐temperature bake renders PEDOT:PSS water‐stable while maintaining its electronic properties (**Figure** **1**a). This heat treatment is sufficient to achieve robust adhesion of films of commercially available PEDOT:PSS to a range of substrates, including glass, gold, parylene‐C, polydimethylsiloxane (PDMS), styrene‐ethylene‐butylene‐styrene (SEBS), and even a range of fabrics without the use of any additives or further pre/post treatments. We attribute this effect to the phase separation of PEDOT and PSS, leading to the formation of linked, PEDOT‐rich insoluble domains. To demonstrate the relevance of this process to bioelectronic devices, we fabricate organic electrochemical transistors (OECTs), stimulating electrodes for the spinal cord, and recording electrodes for electrocorticography (ECoG). Remarkably, devices prepared using heat‐treated PEDOT:PSS show better electrical performance, lower device‐to‐device variability, and similar long‐term stability compared to GOPS cross‐linked films and maintain their performance in vivo. Our approach provides a straightforward method for producing cross‐linker‐free PEDOT:PSS‐based bioelectronic devices that are compatible with existing, widely used fabrication workflows. Finally, we show that spatially controlling the delivery of thermal energy enables new methods of patterning of PEDOT:PSS such as thermal stamping and femtosecond laser‐based heating, allowing us to directly pattern complex, 3D microscale topographies into unmodified PEDOT:PSS films. These straightforward methods to pattern complex conductive polymer devices potentially enable new applications in areas ranging from active nanophotonics^[^ ^10^, ^28^ ^]^ to cell‐device interfaces.^[^ ^29^ ^]^

## 2 Results and Discussion

The baking time required to achieve water‐stable films varies with temperature (Figure 1b), with lower temperatures requiring longer baking times. For example, only 1 min is required to stabilize PEDOT:PSS when baked at 180 °C, whereas 2 min are required at 150 °C. At treatment temperatures below 140 °C, the films do not become water‐stable for treatment times up to 3 min. The process that underlies the modulation of solubility is accelerated by increasing temperature, either by increasing the driving force of the structural transition or by aiding the kinetics of the process by improving mass transport.

We investigated the physical properties of the film before and after heat treatment and subsequent washing with deionized (DI) water using atomic force microscopy (AFM). Following washing, the thickness of heat‐treated PEDOT:PSS films is reduced from ≈ 93 to 46 and 65 nm for treatment times of 1 and 5 min, respectively (Figure S1, Supporting Information). This loss of material indicates that regions of the film that were not sufficiently modified by the heat treatment process are still redispersed upon exposure to water. AFM phase imaging of the film before and after heat treatment reveals differences in film morphology (Figure 1c–e). Phase imaging captures the mechanical properties of the sample surface by monitoring the phase lag between the driving and output signal of an AFM cantilever. It is widely used to map the composition of polymer films. Stiffer regions, which are likely to be richer in PEDOT, show more positive phase lag while softer regions, likely to be PSS rich, exhibit more negative phase lag. The AFM phase images show a coarsening of the microstructure following heat treatment. Relative to the homogeneous distribution in the pristine state, the distribution after heat treatment is shifted toward interleaved but distinct high and low stiffness phases, implying the emergence of separate networks of PEDOT and PSS‐rich domains (Figure 1d). Similar apparent phase separation has been observed after laser‐induced annealing of PEDOT:PSS, where the application of photo‐thermal energy is used to enhance conductivity by connecting PEDOT domains.^[^ ^25^, ^26^, ^30^, ^31^, ^32^, ^33^ ^]^ Subsequent washing results in a marked reduction in the relative area of the softer regions, which are likely to be richer in PSS (Figure 1e).

Measurement of the sheet resistance allows these microstructural changes to be correlated with the electronic transport properties of the films. The sheet resistance decreases upon baking at 180 °C, with longer treatment times resulting in lower resistances (Figure 1f). This conductivity enhancement is also seen for lower (150 °C) and higher (200 °C) temperature heat treatments. The largest increase in conductivity, however, occurs after washing the sample (Figure S2, Supporting Information), which is consistent with the picture that washing results in the elimination of excess electronically insulating PSS. Previously reported treatments to increase conductivity frequently either modify the morphology of PEDOT:PSS to enhance connectivity between isolated PEDOT‐rich regions,^[^ ^13^ ^]^ or remove the insulating PSS component.^[^ ^34^ ^]^

The Raman spectra of heat‐treated PEDOT:PSS films show a blueshift in the bands corresponding to the PEDOT backbone stretch (Figure S3, Supporting Information). This blueshift indicates enhanced linearity and planarity of the PEDOT backbones,^[^ ^35^ ^]^ attributed to increased aggregation in PEDOT‐rich regions. From X‐ray photoelectron spectroscopy (XPS) of the sulfur 2p emission, we observe a decrease in the PSS‐to‐PEDOT ratio at the surface of films that have been heat treated and subsequently washed with DI water (Figure S4, Supporting Information). Mechanical force spectroscopy, which quantifies changes in the stiffness of the film, reveals that the mean of Young's modulus of PEDOT:PSS thin films increases by over 200 MPa after washing (Figure S5, Supporting Information). This suggests that washing may result in the removal of softer, water‐soluble PSS‐rich regions, consistent with previous reports where excess PSS is washed away after chemical cross‐linking to the substrate.^[^ ^27^ ^]^ The combined results from AFM, Raman spectroscopy, XPS, and electrical characterization suggest that heat treatment followed by washing both enriches PEDOT‐rich regions and washes away excess PSS. During the baking step, an insoluble, PEDOT‐rich phase forms a connected network. This PEDOT‐rich phase likely nucleates and grows from the substrate where the heat is applied, leaving the PSS‐rich phase at the top of the film, which can be readily washed off with water. Therefore, we attribute the enhanced conductivity of the heat‐treated films to the removal of excess electrically inactive PSS.

In addition to the widely used and commercially available PH1000 dispersion of PEDOT:PSS, heat treatment also imparts water stability to PEDOT:PSS synthesized in‐house via oxidative polymerization (Figure S6, Supporting Information). This suggests the mechanism relates to energetically favorable transitions occurring at high temperatures that may be common across many PEDOT:PSS formulations, rather than effects specific to additives found in particular blends.

**Figure 2:**
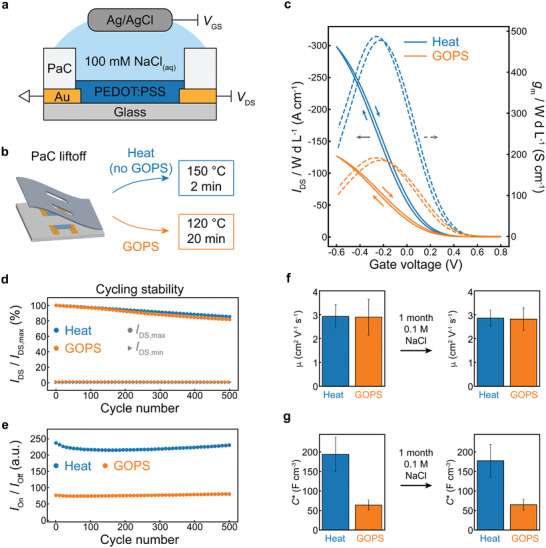
Comparison of heat‐treated and GOPS‐cross–linked organic electrochemical transistors (OECTs). a) Schematic structure of an OECT and b) parylene peel‐off patterning method followed by baking at 150 °C for 2 min and 120 °C for 20 min for the heat‐treated and GOPS‐cross–linked OECTs, respectively. c) Geometry normalized transfer characteristics of heat‐treated (blue) and GOPS‐cross–linked OECTs (orange). d,e) Cycling stability during repeated transfer measurements between *V*_GS_ = 0.2 V to *V*_GS_ = −0.6 V at a scan rate of 160 mV s^−1^. Comparison of f) hole mobility, and g) volumetric capacitance of the heat‐treated (blue) and GOPS‐cross–linked (orange) PEDOT:PSS OECTs before and after soaking in 100 mm aqueous sodium chloride for 1 month. The value and error bars represent the mean and standard error for *n* = 5 OECTs.

We evaluated the performance of the heat‐treated PEDOT:PSS thin films by fabricating typical bioelectronic devices using heat treatment. We started with one of the basic building blocks of bioelectronics: the OECT^[^ ^3^ ^]^ (**Figure** **2**a). To compare heat‐treated and GOPS‐cross–linked PEDOT:PSS as OECT channels, we fabricated devices using a conventional parylene peel‐off method^[^ ^36^, ^37^ ^]^ using PEDOT:PSS dispersions with and without GOPS (Figure 2b). Both the heat‐treated and GOPS‐cross–linked devices were prepared from a blend containing the widely used conductivity‐enhancing additive, ethylene glycol. The improved conductivity imparted by the use of this additive is maintained after heat treatment and washing (Figure S7, Supporting Information). The device without GOPS was subjected to a heat treatment at 150 °C for 2 min, while the GOPS‐containing device was heated for 20 min at 120 °C. The resulting film thicknesses were 41 ± 9 nm for heat‐treated PEDOT:PSS and 134 ± 25 nm for GOPS‐cross–linked PEDOT:PSS. The GOPS‐cross–linked films had a much higher thickness than heat‐treated ones despite using identical spin‐coating parameters (Figure S8, Supporting Information). The higher thickness of GOPS‐cross–linked films is likely due to GOPS immediately starting to react with the sulfonate units of the PSS when added to the dispersion, leading to an increase of the viscosity.^[^ ^19^, ^24^ ^]^

The geometry‐normalized OECT response (Figure 2c) shows a notable increase in the geometry‐normalized drain current for the device using the heat‐treated PEDOT:PSS channel. We also compare the heat‐treated and GOPS‐cross–linked films using several different performance metrics. First, we compare conductivity (σ), a materials property which indicates the resistance of a structure manufactured from a particular material. We also compare the OECT peak transconductance (*g*_m,max_) and threshold voltage (*V*_th_), which are a measure of the ability of the transistor to amplify a small signal and the voltage required to turn on the transistor, respectively.^[^ ^3^ ^]^ These device properties are extracted from the transfer curve in Figure 2c (*V*_D_ = −0.6 V). Finally, we compare the materials properties that determine transconductance,^[^ ^38^ ^]^ the hole mobility (*µ*_h_) and volumetric capacitance (*C* ^*^), which are measured using a previously reported AC characterization method (*V*_G,offset_ = 0.0 V, *V*_D_ = −0.1 V).^[^ ^39^, ^40^ ^]^ For all figures of merit, the heat‐treated PEDOT:PSS OECT either matched or outperformed devices produced using GOPS‐cross–linking. The average and standard deviation of the performance metrics for *n* = 5 devices are summarized in **Table** **1**.

**Table 1:** Performance metrics of OECTs made with heat‐treated and GOPS‐cross–linked PEDOT:PSS channels. Average and standard deviation are computed for *n* = 5 devices.

Channel material	σ [S cm^−1]^	*g*_m,max_ [S cm^−1]^	*V*_th_ [V]	*µ*_h_ at *V*_G_ = 0.0 V [cm^2^ V^−1^ s^−1]^	*C* ^*^ [F cm^−3]^	*V*_gm,max_ [V]	[*µ*_h_ *C* ^*^] from *g*_m,max_ [F cm^−1^ V^−1^ s^−1]^	[*µ*_h_][*C* ^*^] measured independently [F cm^−1^ V^−1^ s^−1]^
Heat‐treated (pristine)	261 ± 73	390 ± 50	0.38 ± 0.02	2.9 ± 0.5	194 ± 44	−0.26 ± 0.02	609 ± 34	563 ± 128
GOPS‐cross–linked (pristine)	114 ± 40	146 ± 42	0.45 ± 0.02	2.9 ± 0.8	64 ± 13	−0.20 ± 0.04	224 ± 28	186 ± 38
Heat‐treated (after 31 days in 0.1 m NaCl_aq_)	254 ± 63	351 ± 64	0.39 ± 0.02	2.9 ± 0.3	188 ± 40	−0.24 ± 0.04	557 ± 43	545 ± 116
GOPS‐cross–linked (after 31 days in 0.1 m NaCl_aq_)	108 ± 43	136 ± 19	0.44 ± 0.01	2.8 ± 0.5	65 ± 14	−0.18 ± 0.05	219 ± 14	182 ± 39

GOPS cross–linking is used to stabilize PEDOT:PSS thin films when exposed to water.^[^ ^19^, ^24^, ^27^ ^]^ Thus, to compare heat‐treated and GOPS‐cross–linked PEDOT:PSS devices, we must assess the stability of the devices when continuously operated in a water‐based electrolyte. To this end, we monitored the stability of OECTs with both heat‐treated and GOPS‐cross–linked PEDOT:PSS channels when cycled from nearly off to on, ensuring to stay within the stability window of the OECTs.^[^ ^41^ ^]^ We monitor both the maximum on‐state current and the on‐off ratio of the devices over 500 electrochemical cycles between *V*_G_ = 0.4 V to *V*_G_ = −0.6 V (Figure 2d,e). We find that the heat‐treated PEDOT:PSS is slightly more stable than GOPS‐cross–linked PEDOT:PSS over 500 cycles, and yields a substantially higher on‐off ratio. This result shows that the heat‐treated PEDOT:PSS film is completely stable during short‐term operation in 100 mm aqueous NaCl, the most common electrolyte in biological systems.

To test the long‐term stability, we also soaked the devices in 100 mm aqueous NaCl for 31 days and tested the performance following soaking (Figure 2f,g). Following the soak, both the heat‐treated and GOPS‐cross–linked PEDOT:PSS films showed nearly identical performance as OECTs (figures of merit summarized in Table 1). This measurement demonstrates that heat‐treated PEDOT:PSS films are insoluble in water and will not suffer from the stability issues one might expect when lacking covalent cross–linking. Overall, the OECT comparison shows that the heat‐treated PEDOT:PSS outperforms GOPS‐cross–linked films in several metrics, is just as stable with short‐ and long‐term exposure to aqueous electrolyte, and reduces both the time and variability of the fabrication process (see standard deviations in Table 1).

To demonstrate the applicability broadly in bioelectronics, heat‐treated PEDOT:PSS thin films were applied in the fabrication of a range of implantable device architectures and tested in vivo. The previously discussed parylene peel‐off (Figure 2b) is the most widely used patterning technique. Using this fabrication method combined with heat treatment of the PEDOT:PSS coatings, we constructed a circumferential spinal cord stimulator (Figure S9, Supporting Information) featuring 32 electrodes distributed in two rows on a flexible substrate.^[^ ^42^ ^]^ On this device, interconnects and electrode sites are 100 nm thick gold and the electrodes are coated with 220 ± 20 nm of PEDOT:PSS. We assessed the performance of the heat‐treated devices by replicating the impedance tests from the literature^[^ ^42^ ^]^ in an awake implanted rat (*N* = 13 electrodes). The average impedance at 1 kHz (Figure S9, Supporting Information) across these electrodes (7.4 ± 1.03 kΩ) is comparable to previously reported devices of the same design 1 to 6 kΩ^[^ ^42^ ^]^ results in phosphate buffered saline (PBS). This impedance study demonstrates the functionality of heat‐treated PEDOT:PSS for acute experiments using standard fabrication techniques.

Dry etching is an emerging and reliable fabrication method to pattern PEDOT: PSS for transparent medical devices.^[^ ^43^ ^]^ To pattern the PEDOT:PSS thin film, the entire wafer is coated and baked, and then an etch mask is patterned with a photoresist on top of the PEDOT:PSS film to define the electrodes and/or tracks. The exposed PEDOT:PSS is etched away with a dry etching process (see Methods), leaving PEDOT:PSS only where the etch mask covers the film. With the dry etching process, the PEDOT:PSS film is coated onto a flat substrate, so the thickness of the resulting films is much more uniform when compared to devices defined with a sacrificial parylene peel‐off method.

**Figure 3:**
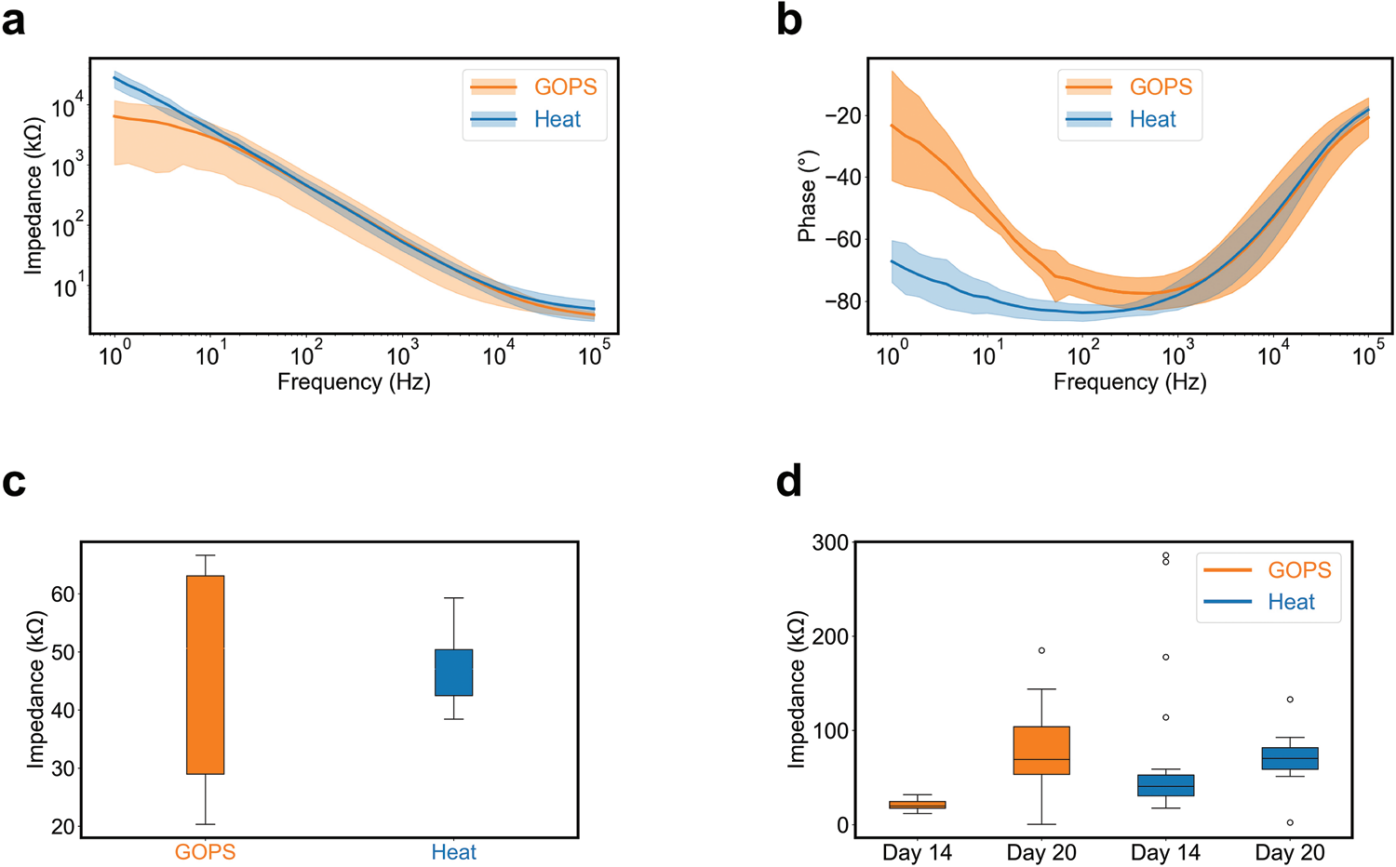
Comparison of heat‐treated and GOPS‐cross–linked laminar implantable probes. Electrochemical impedance spectroscopy (EIS) comparing 10 electrode channels picked at random on 3 different heat‐treated (orange) and GOPS‐cross–linked devices (blue) each showing the device a) impedance, and b) phase response. The solid lines and shaded areas in a) and b) represent the mean and standard deviation, respectively, for measurements from *n* = 10 electrodes. c) Impedance (kΩ) distribution at 1 kHz for the same 10 electrode channels picked at random on 3 different heat‐treated and GOPS‐cross–linked devices each. d) Impedance (kΩ) against frequency graph of fully transparent ECOG device after implantation, after 14 days and 20 days for 10 channels picked at random. The boxes in c) and d) represent the upper and lower quartiles, the whiskers represent the upper and lower extremes, and the line in the box in d) represents the median for measurements from *n* = 10 electrodes.

Using this fabrication method, we constructed a laminar probe design that consists of two multi‐shank legs designed to record deep brain activity. The probe has 32 electrodes (15 µm by 15 µm) distributed in groups of four on each shank. The interconnects consist of 100 nm thick gold and the exposed electrodes are coated with PEDOT:PSS (290 ± 8 nm thick for the GOPS‐cross–linked probes, 220 ± 20 nm thick for the heat‐treated probes). After fabrication, the performance of the heat‐treated devices was compared with the GOPS‐cross–linked devices using electrochemical impedance spectroscopy (EIS) (**Figure** **3**a). The average impedance of both the heat‐treated and GOPS‐cross–linked PEDOT: PSS‐coated electrodes are comparable (47.6 ± 6.5 kΩ and 54.6 ± 33.7 kΩ, respectively), despite the difference in layer thickness. The GOPS‐cross–linked devices show a decrease in the phase of the EIS response at low frequencies (Figure 3b). For Randle's equivalent circuit model, this decrease in phase is associated with a more resistor‐like behavior, usually indicating parasitic charge transfer reactions at the electrode surface.^[^ ^44^ ^]^ For the heat‐treated electrodes, we see only a minimal decrease in the phase response, indicating that they behave more like an ideal capacitor as desired for stimulation electrodes. The lower impedance of the heat‐treated PEDOT:PSS can be attributed to its higher volumetric capacitance. The impedance of 10 channels picked randomly at 1 kHz (Figure 3c) shows an increased variability in electrode impedance for the GOPS‐cross–linked devices compared to the heat‐treated devices, similar to the results for OECTs.

To validate the heat‐treatment method for implantable devices, we tested the stability of heat‐treated PEDOT:PSS coatings in realistic operating environments. A previous study has shown that in accelerated aging environments, the primary mode of failure of thin‐film electrodes is delamination of the metal contact from the substrate.^[^ ^45^ ^]^ Therefore, we compared the performance of heat‐treated and GOPS‐cross–linked PEDOT:PSS coatings in vivo during chronic implantation instead of in a simulated environment. Using dry etching, we manufactured transparent ECoGs which feature 32 electrodes organized as six rows where the outermost rows consist of four electrodes each, and the inner four rows consist of five electrodes each. Using these microscopy‐compatible transparent ECoGs^[^ ^43^ ^]^ and according to a surgical method previously reported,^[^ ^46^ ^]^ female Lewis rats were implanted with bilateral arrays, one with heat‐treated PEDOT:PSS electrodes, and one with GOPS‐cross–linked PEDOT:PSS electrodes (Figure S10, Supporting Information). Despite having almost identical average impedances (Figure 3d), the uniformity of the working electrode impedances at day 14 was much better for GOPS‐cross–linked electrodes (69.6 ± 45.3 kΩ) than heat‐treated electrodes (69.7 ± 75.5 kΩ). At day 20 however, the impedance of working electrodes coated with heat‐treated PEDOT:PSS (69.6 ± 39.7 kΩ) is lower compared to electrodes coated with GOPS‐cross–linked PEDOT:PSS (81.2 ± 45.3 kΩ). While our initial chronic study is limited to just one trial, we have found that the heat‐treated PEDOT:PSS electrodes have comparable long‐term stability in vivo compared to GOPS‐cross–linked PEDOT:PSS electrodes.

To investigate the relevance of our heat treatment method for flexible bioelectronics, we studied the adhesion of heat‐treated PEDOT:PSS to the elastomeric substrates PDMS and SEBS. Spin‐coating PEDOT:PSS films on PDMS and SEBS followed by heat treatment at 180 °C for 2 min showed that the films were water‐resistant (Figure S11, Supporting Information). To evaluate its elasticity and capabilities for flexible electronics, we conducted impedance tests of PEDOT:PSS upon stretching (Figure S12, Supporting Information). The results show that both GOPS‐cross–linked and heat‐treated films can be stretched while maintaining their conductivity, where both samples show a comparable increase in impedance upon stretching.

**Figure 4:**
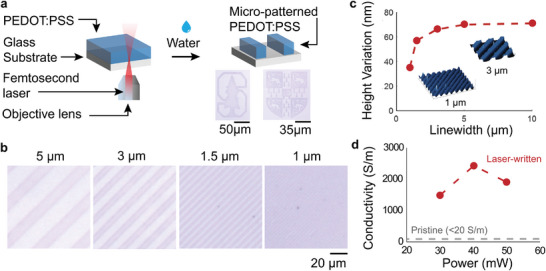
Localized application of thermal energy enables lithography‐free patterning with femtosecond laser processing. a) Schematic of the fabrication process. Thin films of pristine, spin‐coated PEDOT:PSS are exposed to a focused femtosecond pulsed laser beam using the commercial Nanoscribe instrument. After development in water, only the exposed patterns remain. Optical micrographs of patterned Stanford and Cambridge logos measured in reflection are included as insets. b) Optical micrographs of PEDOT:PSS microwires produced via our direct write process. Microwires with linewidths ranging from 5 µm to 1 µm are periodically with a duty cycle of 1/3. c) A modulation transfer function for the writing process, illustrating the variation of achievable contrast with feature size. Neighboring wires begin to impinge on each other as the wire density increases, resulting in reduced contrast for the write condition of 1 µm linewidth and 3 µm periodicity. d) Conductivity of laser‐written PEDOT:PSS structures at different laser powers, measured using the van der Pauw conductivity measurement scheme.

The usage of heat treatment as a method to modify the solubility of PEDOT:PSS films enables new approaches to the patterning of PEDOT:PSS through local delivery of heat. At the macroscale, applying heat through a patterned metal “stamp” allows for the transfer of the stamp pattern onto the PEDOT:PSS film (Figure S13, Supporting Information). At the microscale, we found that exposure to a focused femtosecond‐laser beam using a commercial instrument (Nanoscribe) allowed us to directly write PEDOT:PSS patterns (**Figure** **4**a). We attribute this effect to laser‐induced heating due to the strong absorption of PEDOT:PSS at 780 nm. Laser intensities between 3–4 mW applied at write speeds of 100 000 mm s^−1^ through a 63 × objective, focused through the back of a transparent glass substrate, were optimal for direct patterning of PEDOT:PSS. Below 2.5 mW, the samples aren't sufficiently exposed and still delaminate when exposed to water, whereas, at dosages above 5 mW, the PEDOT becomes white, possibly due to carbonization (Figure S14, Supporting Information). This laser‐induced heating process allows us to write periodic nano‐wire patterns with well‐separated features at spatial frequencies down to 2 µm, below which there is a reduction in contrast (Figure 4b,c). As with heat‐treated PEDOT:PSS, there is a reduction in the thickness of laser‐exposed films after washing (from ≈ 95 nm in the pristine state to ≈ 70 nm post‐wash) attributed to the loss of PSS. Femtosecond‐laser patterning resulted in large increases in conductivity compared to both unexposed PEDOT:PSS and washed, heat‐treated PEDOT:PSS (Figure 4d), suggesting that the rapid kinetics of femtosecond‐laser heating may improve phase separation induced conductivity enhancements. This laser writing process is also compatible with elastomeric substrates; however, the range of the write power is severely limited due to the burning of the substrate at higher intensities (Figure S15, Supporting Information).

**Figure 5:**
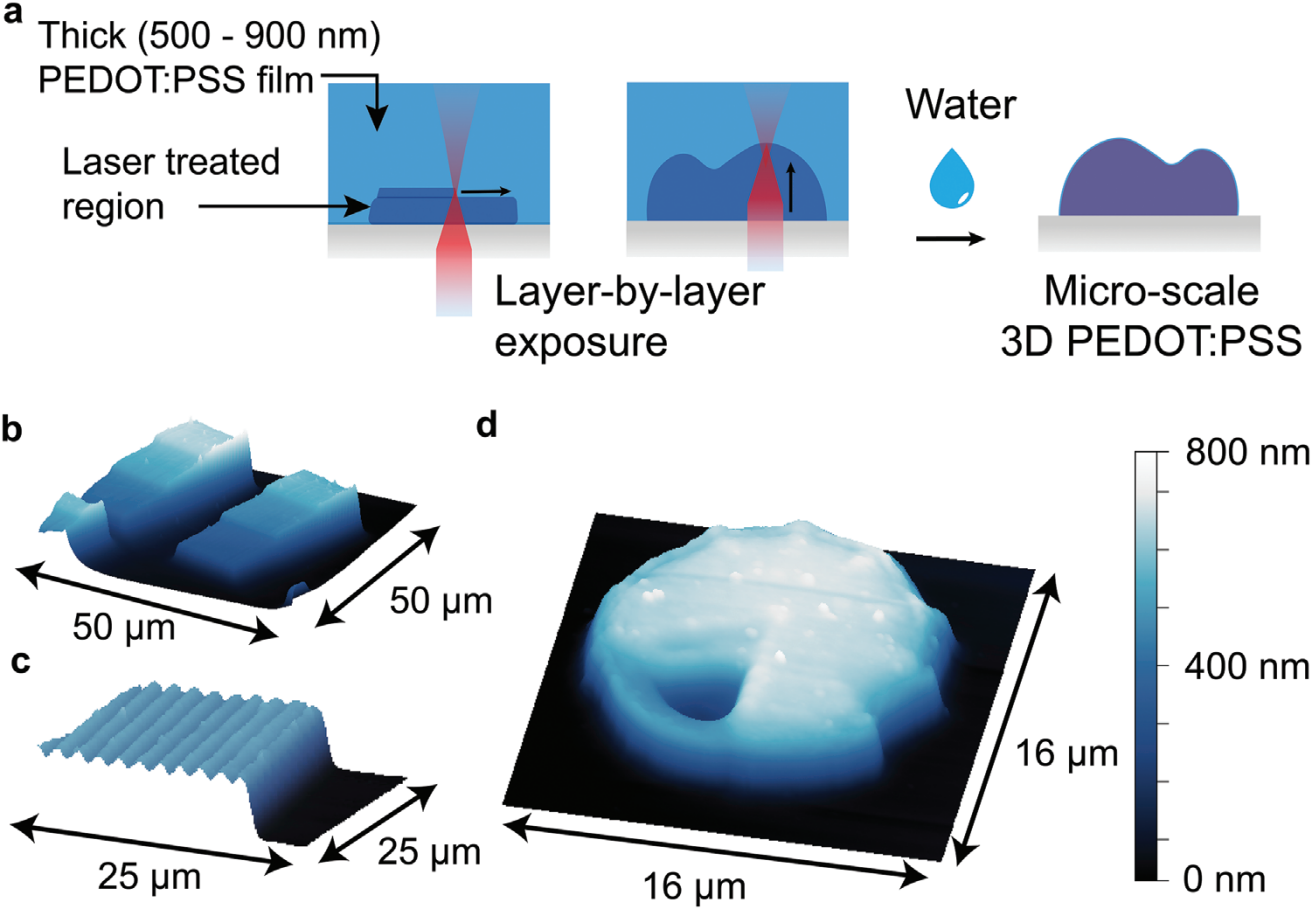
Layer‐by‐layer femtosecond laser patterning of microscale 3D PEDOT:PSS. a)Schematic of the fabrication process. Thick films of pristine, spin‐coated PEDOT:PSS are exposed to a focused femtosecond pulsed laser beam using the commercial Nanoscribe instrument. 3D target patterns are sliced into layers and sequentially exposed upon translation of the laser‐focusing plane with a piezoelectric stage. After development in water, only the exposed patterns remain. Atomic force microscope (AFM) images of b) blocks with different height steps c) a block with a sinusoidal texture written on its top surface, and d) a sculptural test piece with features such as curves, bevels, recesses, and grooves.

The laser patterning method can be extended to 3D using the precise control of focal height offered by the commercial Nanoscribe tool, enabling patterning of PEDOT:PSS surface topographies (**Figure** **5**a). We expose layers with different 2D patterns on different planes, allowing complex, multi‐height geometries to be constructed. We demonstrate these capabilities with a test pattern featuring blocks of varying heights (Figure 5b), a block micropatterned with a sinusoidal surface texture (Figure 5c), and a sculptural test piece with features such as curves, bevels, recesses, and grooves (Figure 5d). Unlike grayscale lithography, which creates multi‐height features by modulating the dose applied to a 2D pattern, our technique allows for the scanning of a focal spot with three degrees of freedom in *x, y*, and *z*, more closely resembling 3D printing. Further discussion on the considerations for femtosecond laser patterning of 3D PEDOT:PSS structures can be found in Note S1, Supporting Information. We expect this 3D patterning method will enable a new level of control over the geometry of functional PEDOT:PSS microstructures.

## 3 Outlook

Despite the drawbacks of chemical cross‐linking of PEDOT:PSS, it has been considered a necessity to achieve water‐stable films for applications in bioelectronics. For manufacturing, heat‐treated PEDOT:PSS offers a quicker and equally reliable method delivering similar or better performance in bioelectronic devices. In OECTs, heat‐treated PEDOT:PSS films significantly outperform GOPS‐cross–linked devices due to their larger volumetric capacitance and have the same stability when left in aqueous electrolyte for a one‐month period. For implantable electrodes, both heat‐treatment and GOPS‐cross–linking offer consistently the same order of magnitude in impedances across different batches and device designs. In chronic implantations, the heat‐treated PEDOT:PSS coatings showed comparable performance and stability as GOPS‐cross–linked ones. This technique also suppresses the risk of GOPS reacting upon exposure to air and moisture which then affects the quality of the cross–linking, therefore increasing impedance over time. This heat treatment process greatly simplifies the device fabrication process by avoiding chemical additives whose quality varies with air exposure, often introducing substantial device‐to‐device and batch‐to‐batch variance. This heat‐treatment method is also applicable to surfaces with diverse surface chemistries, including glass, parylene, PDMS, SEBS, gold (Figure S16, Supporting Information), and even household fabrics (Figure S17, Supporting Information), highlighting the broad applicability of this approach.

Furthermore, the flexibility of heat treatment opens the door to new methods of directly patterning PEDOT:PSS by spatially controlling the delivery of thermal energy at many length scales. Low‐cost and scalable patterning of large‐area films could be achieved by applying heat through thermal stamping approaches that are compatible with roll‐to‐roll manufacturing. For micropatterning, high‐resolution patterns with micron‐scale feature sizes are achievable using a femtosecond laser. This method achieves comparable resolution to previous methods which use alternate chemistries or photosensitive additives,^[^ ^47^, ^48^, ^49^ ^]^ but requires only commercially available PEDOT:PSS along with established two‐photon laser patterning tools, facilitating broad adoption. While detailed characterization of the properties of films prepared using these methods is required to validate their performance, these direct‐write approaches could help simply device fabrication workflows. The ability to directly pattern PEDOT:PSS at the microscale in 3D could also enable emerging applications ranging from complex electroactive cell cultures to nanophotonics, where performance is dictated by controlling the geometry of functional materials at the microscale. These applications may be aided by future work that explores the writing of larger, higher aspect ratio structures through process optimization and alternative film treatments. At a fundamental level, operando techniques such as ultra‐fast X‐ray diffraction could allow the dynamics of microstructural evolution from slow heating to be compared with ultra‐fast heating from femtosecond laser exposure to provide further insights into the thermodynamics and kinetics of heat‐assisted phase separation of PEDOT:PSS.

## 4 Experimental Section

### Preparation of Conducting Polymer Solutions

The commercial PEDOT:PSS dispersion, Clevios PH1000 (Heraeus), was used to prepare samples from unmodified PEDOT:PSS. An ethylene glycol‐containing dispersion was prepared by mixing Clevios PH1000 with 6%v/v ethylene glycol and sonicating for 10 min to mix. 1%v/v of (3‐glycidyloxypropyl)trimethoxysilane (GOPS) was added to the GOPS‐cross–linked PEDOT:PSS solutions, and then both solutions were sonicated again for 2 min. Before spin coating, the solutions were filtered through a 0.45 µm polytetrafluoroethylene (PTFE) syringe filter.

For the spin‐coating of thick, uniform PEDOT:PSS layers, a viscous, high solid content PH‐1000 dispersion was prepared by reducing the solvent volume with rotary evaporation. An open beaker with 20 mL PH 1000 was heated at 85 °C for 1 h while stirring with a magnetic stir bar at 500 rpm. The dispersion was sonicated for 10 min and filtered through a 0.45 µm syringe filter before spin coating.

### Atomic Force Microscopy Characterization

Tapping‐mode AFM images were collected in the air using an AFM Asylum MFP‐3D. NSC35/Cr‐Au cantilevers (MikroMasch) with a force constant of 5.4 N m^−1^ were used. For height measurements (Figure 4), images with maximum dimensions of 90 µm were collected with scan rates of 1 Hz. For phase measurements, images with dimensions of 1 µm were collected at scan rates of 0.5 Hz. Images were processed with Gwyddion.

### Conductivity Measurements

The sheet resistance of the PEDOT:PSS films in Figure 1f and Figure S7 (Supporting Information) were measured using a four‐point probe tool (Ossila). Samples were prepared from PEDOT:PSS formulations spin‐coated onto glass substrates. The sheet resistance of finite structures was extracted using established empirical size corrections.^[^ ^50^ ^]^ Samples in Figure 4d and Figure S2 (Supporting Information) were measured with van der Pauw measurements using an Agilent Semiconductor Analyzer, with measurements taken across gold contact pads at the corners of square PEDOT:PSS samples.

### Fabrication of Conducting Organic Electrochemical Transistors

Organic electrochemical transistors (OECTs) for device characterization were fabricated as previously reported.^[^ ^51^ ^]^ Fabrication started with 10 min of sonication of borosilicate glass wafers (900 µm thick, double‐side polished Microchemicals) submerged in acetone followed by IPA then baked at 150 °C to remove any residual moisture. Gold contacts were patterned using metal lift off which consisted of coating with AZ nLoF 2035 negative photoresist (Microchemicals) (spin coating at 500 rpm for 5 s, acceleration of 1000 rpm s^−1^, followed by 3000 rpm for 45 s, acceleration of 8000 rpm s^−1^, then soft baked at 110 °C for 60 s) followed by UV exposure (60 mJ cm^−2^), a post exposure bake (110 °C for 180 s), and development in AZ 826 MIF (Microchemicals) for 30 s. The patterned wafer was coated with 5 nm titanium and then 100 nm gold (E‐beam Evaporator, Kurt J. Lesker Company), and lift‐off was performed by submerging the wafer in NI555 stripper (Microchemicals) for 10 hours followed by rinsing with acetone then IPA. The patterned metal‐coated wafers were then coated with a parylene bilayer by first treating the wafer with oxygen plasma for 60 s followed by submerging the wafer in a dilute silane solution (3%v/v A174 silane dissolved in 0.1%v/v acetic acid in deionized water) for 45 s to improve parylene adhesion to the wafer. The silane‐treated wafer was rinsed with ethanol and heated for 1 h at 75 °C then coated with a 2 µm layer of parylene (PDS 2010 Labcoater 2, Specialty Coating Systems) followed by coating with a soap surfactant layer (2%v/v/ Micro 90 soap in deionized water spin coated at 1000 rpm for 30 s and dried in air for 20 min) followed by a second deposition of a 2 µm layer of parylene. The trenches for depositing polymer channels were defined in the parylene bilayer with photolithography by coating with AZ10XT positive resist (Microchemicals) (spin coating at 3000 rpm for 45 s, acceleration of 8000 rpm s^−1^, soft bake at 115 °C for 120 s) followed by UV exposure (540 mJ cm^−2^) and developing in AZ 726 MIF developer (Microchemicals) for 10 min. Then, trenches were etched using reactive ion etching (recipe) and the wafers were diced with a diamond scribe and tile cutter tool. Conducting polymers were coated using the same spin coating parameters described above. The conducting polymer channel was defined by peeling off the top parylene layer using Kapton tape, leaving the conducting polymer only in the patterned trench. The GOPS‐cross–linked PEDOT:PSS OECTs were annealed at 120 °C for 20 min. The heat‐treated PEDOT:PSS OECTs were annealed at 150 °C for 2 min. A silicone well was defined using an adhesive‐backed silicone (McMaster‐Carr) to confine the electrolyte.

### Fabrication of Electrodes for Electrocorticography

Four inches wafers (500 µm thick) were rinsed in acetone, and isopropyl alcohol (IPA) and then baked at 150 °C for 3 min. After dehydration baking, they were coated with 2 µm of parylene (SCS, Labcoter 2, PDS 2010). Then, to pattern metals, they were spin‐coated with 3.4 µm of negative lift‐off photoresist AZnLOF 2035 (Microchemicals GmbH, Germany) (spin coating at 500 rpm for 5 s, acceleration of 1000 rpm s^−1^, followed by 3000 rpm for 30 s, acceleration of 3000 rpm s^−1^, then soft baked at 110 °C for 60 s). They were exposed to ultraviolet (UV) light using a mask aligner (MA/BA 6, Suss MicroTec, Germany) (with a dose of 90mJ cm^−2^). They were baked post‐exposure at 105 °C for 60 s and then developed in AZ726MIF (Microchemicals GmbH, Germany) for 90 s. The patterned wafer was coated with 10 nm titanium and then 100 nm gold (E‐beam Evaporator, Kurt J. Lesker Company), and lift‐off was performed by submerging the wafer in acetone overnight followed by rinsing with acetone and then IPA.

For devices with organic layers patterned directly with photolithography, the PEDOT:PSS formulation was spin‐coated (1500 rpm, 5000 rpm s^−1^ acceleration for 5 s) and the film was baked for 1 h at 110 °C for the GOPS‐cross–linked devices and 2 min at 180 °C for the heat‐treated devices. It was then soaked 11 min in DI water three times with DI water rinse in between each puddle to remove excess PSS and low molecular weight compounds. A photoresist etch mask (2.2 µm thick) was lithographically defined with the photoresist AZ 5214E (Microchemicals GmbH, Germany). It was soft‐baked at 110 °C for 60 s, UV exposed at 150 mJ cm^−2^, and then developed in AZ726MIF for 50 s. The PEDOT: PSS layer was subsequently etched by reactive ion etching using CF_4_ and O_2_ (5 sccm and 50 sccm flowrates respectively, at 60 mTorr pressure and 150 W power using a Plasma Pro80 RIE, Oxford Instruments) during 2 min 45 s for the GOPS‐cross–linked devices and 1 min 20 s for the heat‐treated devices. The remaining resist was washed off by an acetone rinse, followed by an IPA rinse. After rinsing the remaining photoresist with acetone, a self‐assembled monolayer (SAM) of the adhesion promoter methacryloxypropyl trimethoxysilane (A 174 Silane, Sigma‐Aldrich, UK) was deposited to improve the adhesion of the PaC insulation layer to the substrate layer of PaC by first treating the wafer with oxygen plasma for 60 s followed by immersing the wafer in a dilute silane solution (3%v/v A174 silane dissolved in 0.1%v/v acetic acid in deionized water) for 30 s. The silane‐treated wafer was rinsed with ethanol and heated for 1 h at 75 °C then coated with a 2 µm layer of parylene. Then, an outline etch mask was patterned using AZ10XT (Microchemicals GmbH, Germany) spin‐coated at 500 rpm for 5 s, 300 rpm s^−1^ acceleration, 3000 rpm for 30 s, 2000 rpm s^−1^ acceleration. The wafer was then soft baked at 110 °C for 120 s, UV exposed at 600 mJ cm^−2^, and developed in 3:1 DI water to AZ400K (Microchemicals GmbH, Germany) for 2 min 30 s. Then the wafer was put in the etcher for 7 min 30 s. After etching, the remaining resist was washed off in acetone and IPA. Using the exact same process, the wafer was patterned with 10XT for the insulation etch. It was then etched for 3 min 45 s using the same recipe as previously. The etch rate of PaC was determined in prior experiments and was used to control its etching to avoid oxidation and damage to the underlying PEDOT: PSS layer. Residues of the etch mask were removed by rinsing with copious amounts of acetone, followed by rinsing with isopropyl alcohol and DI water.

For devices patterned using a sacrificial parylene peel‐off layer, after rinsing of the remaining photoresist with acetone, a SAM of the adhesion promoter methacryloxypropyl trimethoxysilane (A174 Silane, Sigma‐Aldrich, UK) was created to improve the adhesion of the PaC insulation layer to the substrate layer of PaC by bilayer by first treating the wafer with oxygen plasma for 60 s followed by immersing the wafer in a dilute silane solution (3%v/v A174 silane dissolved in 0.1%v/v acetic acid in deionized water) for 30 s to improve parylene adhesion to the wafer. The silane‐treated wafer was rinsed with ethanol and heated for 1 h at 75 °C then coated with a 2 µm layer of parylene. Then, an outline etch mask was patterned using AZ10XT (Microchemicals GmbH, Germany) spin‐coated at 500 rpm for 5 s, 300 rpm s^−1^ acceleration, 3000 rpm for 30 s, 2000 rpm s^−1^ acceleration. The wafer was then soft baked at 110 °C for 120 s, UV exposed at 600 mJ cm^−2^, and developed in 3:1 DI water to AZ400K (Microchemicals GmbH, Germany) for 2 min 30 s. Then the wafer was put in the etcher for 7 min 30 s. After etching, the remaining resist was washed off in acetone and IPA. Three layers of DI water were spin‐coated with 3% soap (1000 rpm, acceleration of 500 rpm s^−1^, 30 s) and left the wafer to dry in air for 5 min between each coating. The wafer was coated with another 2 µm layer of parylene‐c. Using the exact same process for 10XT, the wafer was patterned for the insulation etch. It was then etched for 3 min 45 s using the same recipe as previously. The residues of the etch mask were removed by rinsing with acetone, followed by rinsing with isopropyl alcohol. The wafers were then spin‐coated with the PEDOT:PSS formulation (1500 rpm, 5000 rpm s^−1^ acceleration for 5 s), PEDOT:PSS was carefully wiped off the pads using a swab filled with DI water and the film was baked for 2 min at 110 °C. Following this short bake, the sacrificial layer of parylene‐c was removed after which the GOPS‐cross–linked and heat‐treated devices were baked for one hour at 110 °C and for 2 min at 180 °C, respectively. All devices were then immersed in DI water for 11 min three times with DI water rinse in between each puddle in order to remove excess PSS and low molecular weight compounds.

### Chronic ECOG Implantation Surgical Method

All animal procedures were conducted in full compliance with the UK Animals (Scientific Procedures) Act 1986 and received approval from both the United Kingdom Home Office (project license number PFF2068BC) and the Animal Welfare and Ethical Review Body of the University of Cambridge. Female Lewis rats, weighing 150 to 250 grams (Charles River), were used for this study. All surgical interventions were performed by a single surgeon, S. El Hadwe (I54111393), under isoflurane anesthesia. Body temperature was meticulously maintained throughout the procedure using a thermal blanket.

For device implantation, a midline incision was made along the skull, and the skin was retracted to expose the underlying connective tissue. The skull was carefully exposed with a curette until the sagittal, coronal, and lambdoid sutures were fully visible. Lateral exposure was minimized to protect the temporalis muscles. Bilateral craniectomies, positioned lateral to the sagittal sutures, were created using a drill with a diamond burr. A modified 27‐gauge needle was employed to open the dura, revealing the underlying cortex. Devices were placed over the cortex and initially secured using cyanoacrylate‐based glue, with further stabilization achieved through the application of acrylic cement. A small metallic screw was inserted into the posterior fossa, beneath the lambda, in contact with cerebrospinal fluid, serving as a grounding connection. Both the back end flexible flat cable (FFC) and the grounding wire were tunneled subcutaneously through a pocket over the cervical spine. The incision was closed with 5‐0 polyamide sutures.

Post‐surgery, the animals were allowed to recover and were administered analgesics (Meloxicam, Carprofen) for two days following implantation, in addition to pre‐operative analgesia. The rats were housed in groups of three or four, with ad libitum access to food and water. Electrophysiological recordings were obtained under terminal anesthesia up to 20 days post‐implantation.

The FFC of the implanted device was exposed and connected to a 32‐channel RHS head stage and RHS Stim/Recording Controller (Intan Technologies, USA) for electrophysiology acquisition and stimulation. Grounding was facilitated via the implanted wire, and recordings were acquired for up to 30 min.

### Spinal Cord Implantation Surgical Method

A 4 cm incision was made from T11 to L1, using the last palpable rib as a landmark to expose the thoracolumbar spine. A laminectomy was performed at the T12‐L1 level, exposing the dorsal epidural surface of the thoracic spinal cord. The vertebral pedicles were resected with a rongeur down to the ventral base of the pedicle, providing access to the ventral epidural plane. A blunt dissector was then used to develop this plane toward the contralateral pedicular base, facilitating the passage of the blunt end of a 7‐0 polypropylene suture. The suture was attached to the leading end of the electrode array, and gentle traction was applied to guide the array across the ventral epidural plane.

The selected device was secured using a double‐ended 7‐0 polypropylene suture (Ethicon Inc., Raritan, NJ, USA) to pull it under the spinal cord. To avoid iatrogenic dural injury, the needle was inserted bluntly into the ventral epidural plane, with care taken to guide its tip against bone to avoid encroachment on the spinal cord. After passing the suture, the blunt end was retrieved, and the procedure was repeated with the opposite end to gently pull the device across the ventral spinal cord, ensuring the device did not twist. Once the device was fully in place, its end was tucked under itself over the dorsal spinal cord, providing circumferential coverage. The electrode positioned along the midline of the spine was used to confirm the device's rotational orientation. The device was then secured in place using silicon sealant (Kwik‐Sil, World Precision Instruments, Hitchin, UK).

### In Vitro Electrochemical Impedance Spectroscopy

The impedance of the electrodes was characterized by an Electrochemical Impedance Spectroscopy (Autolab Potentiostat, Metrohm AG, Switzerland) with a platinum electrode as the counter electrode. A sinusoidal voltage input of amplitude 10 mV at different frequencies ranging from 1 to 100 kHz was used for the purpose.

### In Vivo Electrochemical Impedance Spectroscopy

For neural recordings, the brain screw was used as the reference electrode and was connected to the ground of the recording hardware. The RHD USB interface board and software (Intan Technologies, USA) were used for data acquisition (30 000 Hz sampling rate). The head stage was a RHD2164 amplifier chip.

### Direct Femtosecond Laser Patterning

Different substrates and spin coating conditions were used to prepare for patterning standard PEDOT:PSS thin‐films, patterning thick samples and measuring the electrical conductivity of the laser‐patterned PEDOT:PSS. For process development, #1 glass cover slides (VWR, thickness: 150 ± 10 µm, refractive index: 1.53) were spin‐coated with unmodified PEDOT:PSS solution at various speeds 2000 rpm for 1 min, which resulted in a uniform film with a thickness of 90 nm. For thick samples, a previously described high‐solid content, viscous PEDOT:PSS dispersion was spin‐coated at 600/800/1000 rpm for 3 min. For characterizing electrical conductivity, a silicon wafer with 1 µm of thermally grown silicon oxide was first patterned with van der Pauw contacts using photolithography. The substrate was then spin‐coated with unmodified PEDOT:PSS solution at 2000 rpm for 1 min. In all cases, the substrates were cleaned by ultrasonication with IPA and acetone, followed by 10 min of UV ozone treatment.

Direct femtosecond laser patterning was conducted using the Nanoscribe Photonics GT (“Nanoscribe” for short, Nanoscribe GmbH & Co. KG). For patterning process development, a 63× objective (Plan‐Apochromat, 63×/1.4, Carl Zeiss) was used in oil (Immersol 518 F, Carl Zeiss) immersion mode to focus the laser on to the substrate. The PEDOT:PSS‐coated glass substrates were first mounted on the supplied DiLL sample holder and then loaded with the non‐coated side facing the objective for the patterning process. Using the built‐in automatic interface finding functionality of the Nanoscribe, the interface between the substrate and the PEDOT:PSS was found. This allowed the laser to precisely treat the film at the interface, adhering the patterned features to the substrate. To account for potential error in the position of the automatically found interface, the patterning was set to start at various distances ranging from 0.2 to 1.6 µm into the substrate. To pattern the structure used for electrical conductivity measurement, an air‐immersion 20× objective (Epiplan‐Neofluar, 20×/0.5, Carl Zeiss) was used instead, and the substrate was mounted onto the holder with the coated side facing the objective. As the laser spot size focused with the 20× objective was larger than that with the 63× objective, the PEDOT:PSS‐substrate interface was found manually by shifting the laser focal point in the vertical direction until the focal point became barely observable on the built‐in camera of the Nanoscribe.

The digital files containing the design of the features were processed into Nanoscribe‐readable formats by the DeScribe software provided by Nanoscribe. The parameters used for the conversion were as follows: hatching distance – 0.1 µm, slicing distance – 0.1 µm). Using the NanoWrite software supplied by the Nanoscribe, the laser power and scan speed were set to 3.5 mW and 10 000 µm sec^−1^ when using the 63× objective, and 30–50 mW and 5000 µm s^−1^ when using the 20× objective. The sample containing the patterned features was then developed in deionized water for 1 min with periodic and gentle agitation and then dried with N_2_.

### UV–Vis Spectroscopy

A spectrometer (Acton 2300i, SpectraPro) with a cooled charge‐coupled device (CCD) (Princeton Instruments, PIXIS 1024) was used to acquire the transmittance spectra in Figure S4, Supporting Information. A halogen lamp was used for broadband white‐light illumination. The spectra were normalized by subtracting the CCD dark counts from the experimental spectrum, then the difference was divided by the spectrum of the halogen lamp collected via a bare glass substrate used as a reference.

### XPS Measurements

XPS analysis was performed using a Thermo Scientific Escalab 250Xi system with a monochromatic Al kα X‐ray source (1486.7 eV). The X‐ray beam size was set to 650 µm, with data acquired at a pass energy of 20 eV at a step size of 0.1 eV. Electronic charge neutralization was achieved using an ion source, operated at an Ion gun current of 100 µA and a gun voltage of 40 V. All sample data was collected at a pressure below 10^−8^ Torr and a room temperature.

### Raman Measurements

Raman measurements were carried out using a Renishaw inVia Raman confocal microscope with a 633 nm excitation laser under ambient conditions. Emission was collected using a 20× long‐working‐distance objective lens in streamline mode and dispersed by a 1200 l mm^−1^ grating with 5% of the laser power (<25 µW).

### Synthesis of In‐House PEDOT:PSS Dispersions

In‐house PEDOT:PSS was synthesized as previously reported.^[^ ^52^ ^]^ A 250 mL round‐bottom flask was filled with 100 mL of DI water, 0.87 g of poly (sodium 4‐styrenesulfonate) (NaPSS, Mw ≈ 70 000 g mol^−1^) and stirred at 600 rpm for 5 min at room temperature until a clear, viscous solution was formed. Then, 0.65 g of ammonium persulfate (𝑁𝐻_4_)_2_𝑆_2_𝑂_8_ was added (1.5 equivalents with respect to the weight percentage of monomer), followed by a catalytic amount of iron (III) chloride hexahydrate (𝐹𝑒𝐶𝑙_3_⋅6𝐻_2_𝑂). Finally, 0.43 g of 3,4‐ethylenedioxythiophene (EDOT) was added to the solution, resulting in a dispersion with a solid content of 1.3 wt.%. After 2 h, the dispersion turned a dark blue color. It was stirred until complete conversion for 48 h. Subsequently, the dispersions were placed in a 12 kDa dialysis bag and dialyzed for two days in DI water, changing the water every 2–4 h.

### Fabrication of Stretchable PEDOT:PSS on PDMS

PDMS (SYLGARD 184) was spin‐coated on a Nafion 117 (CAS 31175‐20‐9) coated glass slide at 1000 rpm for 60 s. Then, PEDOT:PSS was spun for 5 s, 5000 rpm s^−1^, 1500 rpm. A 4 cm long by 2 cm wide rectangular shape was laser cut and lifted from the carrier.

## Conflict of Interest

The authors declare no conflict of interest.

## Supporting information



Supporting Information

## Data Availability

The data that support the findings of this study are available from the corresponding author upon reasonable request.
