# Supplementary material for: Thermal Processing Creates Water‐Stable PEDOT:PSS Films for Bioelectronics
Source: Adv Mater. 2025 Mar 3;37(13):2415827. doi: 10.1002/adma.202415827 (PMC11962680; doi:10.1002/adma.202415827)
Supplement: Supplementary file 1 — Supporting Information [file ADMA-37-2415827-s001.docx]

**Supplementary Materials for**

**Thermal Processing Creates Water-Stable PEDOT:PSS Films for Bioelectronics**

Siddharth Doshi, Margaux Forner *et al.*

Corresponding Authors: Siddharth Doshi, Nicholas A. Melosh, George G. Malliaras, Scott T. Keene

Corresponding author emails: [sdos@stanford.edu](mailto:sdos@stanford.edu), [nmelosh@stanford.edu](mailto:nmelosh@stanford.edu), [gm603@cam.ac.uk](mailto:gm603@cam.ac.uk), [sk251@rice.edu](mailto:sk251@rice.edu)

**Supplementary Note 1:** *Considerations for 2D and 3D patterning PEDOT:PSS with a femtosecond laser.*

Focusing the laser through the back of the transparent substrate allowed for the use of a high-magnification oil-immersed objective while avoiding contact between the PEDOT:PSS film and immersion oil. Films on substrates that are non-transparent or partially transparent can be patterned by using lower numerical aperture objectives that directly focus the laser beam onto the film through air. The effective heat dose depends on the choice of objective and the heat transfer characteristics of the substrate, necessitating optimization of parameters such as laser power and write speed for each individual experimental configuration. For example, the process window for elastomeric substrates written using a 20 $\times$ air objective at a write speed of 5,000 mm s^-1^ is limited to a narrow 18-20 mW range due to burning of the substrate at higher intensities (**Fig. S15**).

Femtosecond-laser patterning resulted in large increases in conductivity compared to both unexposed PEDOT:PSS and washed, heat-treated PEDOT:PSS (**Fig. 4d**). We determined the conductivity by combining van der Pauw measurements of the sheet resistance with AFM measurements of the thickness of samples that were patterned onto insulating substrates with lithographically prepared Au contact pads (**Fig. S18**). The highest conductivity, of > 2000 S m^-1^, was observed at an applied power of 40 mW (for a configuration that used a 20$\times$ air objective and a write speed of 5,000 mm s^-1^). This is two orders of magnitude higher than that of the pristine control PEDOT:PSS sample (19 S m^-1^, consistent with previous reports) and many times higher than heat-treated and washed PEDOT:PSS samples. This suggests that the rapid kinetics that are expected to be associated with the femtosecond-laser heating may improve phase separation induced conductivity enhancements.

Thicker films were micro-patterned by femtosecond-laser exposure to realize structures with aspect ratios up to ~0.1. Periodic nanowire features with a thickness of 400 nm were cleanly written with feature sizes down to 5 µm. At smaller linewidths, features partially de-bond from the substrate upon development in water, likely due to large swelling-induced stresses overcoming substrate adhesion (**Fig. S19**). The patterning of thicker structures crucially depends on the preparation of viscous, high solid-content PEDOT:PSS dispersions by rotary evaporation of commercially available solution (Clevios PH1000). This allowed for the preparation of thick, 150 – 1000 nm films of PEDOT:PSS in a single spin coat. Samples prepared through sequential spin-coating of multiple thin PEDOT:PSS layers could not be successfully written, likely due to interfacial de-bonding between the sequentially coated layers, nor could drop-casted PEDOT:PSS.


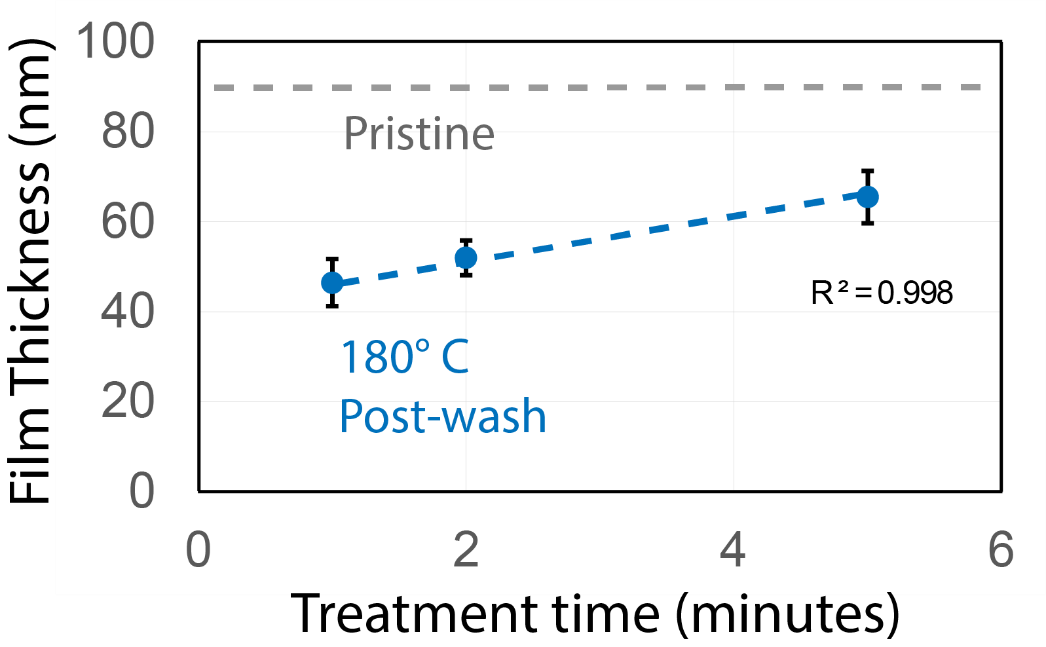


**Figure S1**: **Atomic force microscopy measurements of the thickness of heat treated PEDOT:PSS films after being washed in water**. Films were baked at 180 °C for various treatment times. Measurements are plotted as the mean ± standard deviation (n = 3 measurements at different locations on a single sample). A linear trendline (blue) is shown. The pristine, untreated film thickness is included as a grey dashed line.


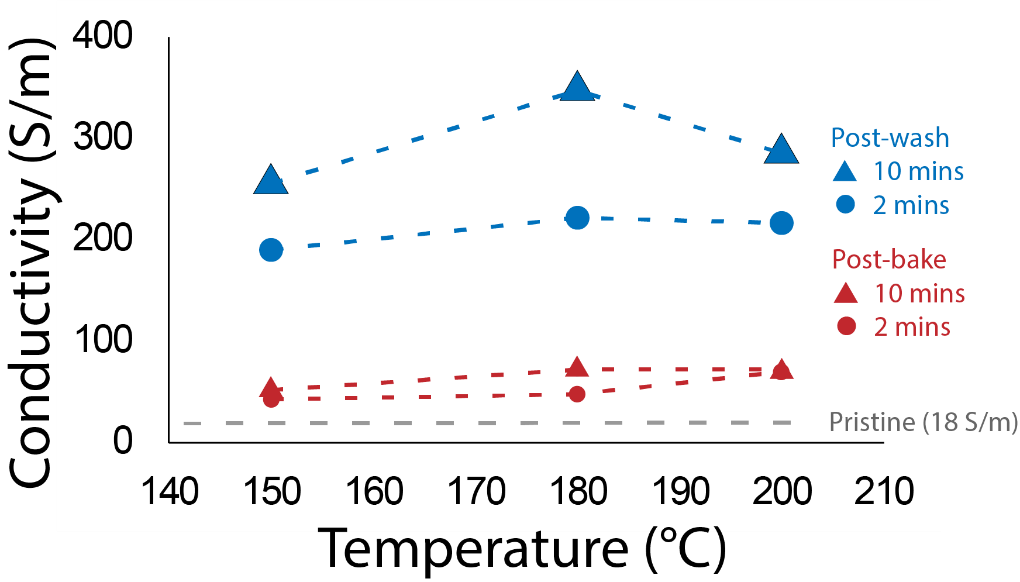


**Figure S2**. **Conductivity of heat treated PEDOT:PSS films across a broad temperature and time window.** Effect of treatment time and temperature on the conductivity of PEDOT:PSS films, both prior to and after exposure to water. The films had a pre-wash thickness of ~ 90 nm. The conductivity was calculated using the sheet resistances measured with the van der Pauw method, on square samples with gold contact pads at their periphery, and film thicknesses measured with a profilometer.


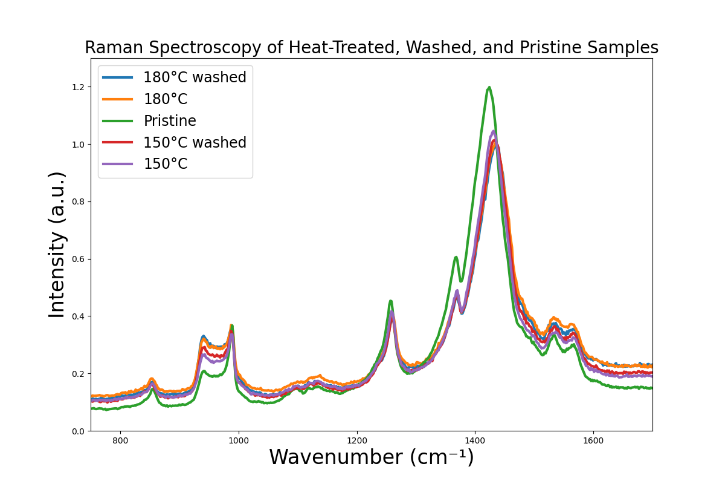


**Figure S3. Raman spectroscopy of PEDOT:PSS films following different heat treatments.** Heat treatment of the PEDOT:PSS film results in a blueshift of the bands corresponding to the PEDOT backbone at *ca.* 1423 cm^-1^ to *ca.* 1434 cm^-1^. The magnitude of the blueshift increases as the temperature is increased from 150 °C to 180 °C and following washing. This blueshift indicates enhanced linearity and planarity of the PEDOT backbones.^[1]^


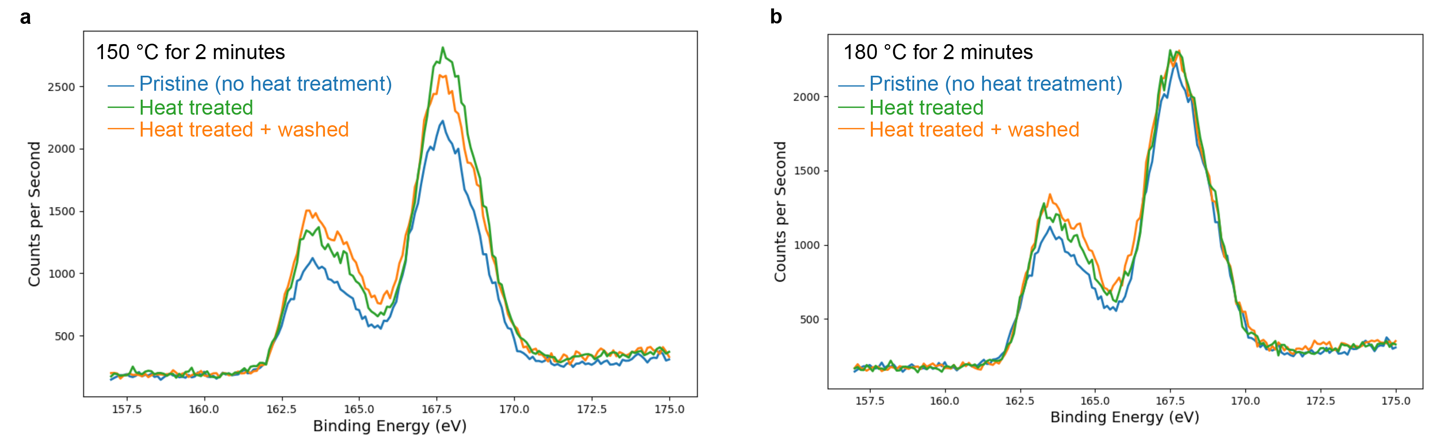


**Figure S4. X-ray photoelectron spectroscopy of heat-treated PEDOT:PSS films.** Following heat-treatment and washing, there is an increase in the relative signal from the sulfur 2p (S2p) peak corresponding to PEDOT (162 eV to 166 eV) relative to the S2p peak from PSS (166 eV to 172 eV) for treatments at both **a**, 150° C and **b**, 180 °C. Using a previously reported method,^[2]^ we used the area under the peaks to extract the ratio of PSS to PEDOT in the film. The pristine sample shows a 2:1 ratio of PSS to PEDOT, while 150 °C shows ratios of 2:1 before washing and 1.8:1 after washing, and 180 °C shows ratios of 1.9:1 before washing and 1.8:1 after washing, indicating that excess PSS is washed away and PEDOT is enriched in the heat-treated films.





**Figure S5**. **Mechanical properties of heat-treated PEDOT:PSS films**. Effect of baking for 2 minutes at 180 °C on the stiffness of 100 nm thick PEDOT:PSS films, both prior to and after exposure washing. The Young’s modulus was measured using force volume spectroscopy with a specialized colloidal atomic force microscopy (AFM) tip. Measurements were made at 64 points over a 50 µm^2^ area on a single sample for each condition. The modulus distribution for each condition was fitted to generate the plotted force volume curves.


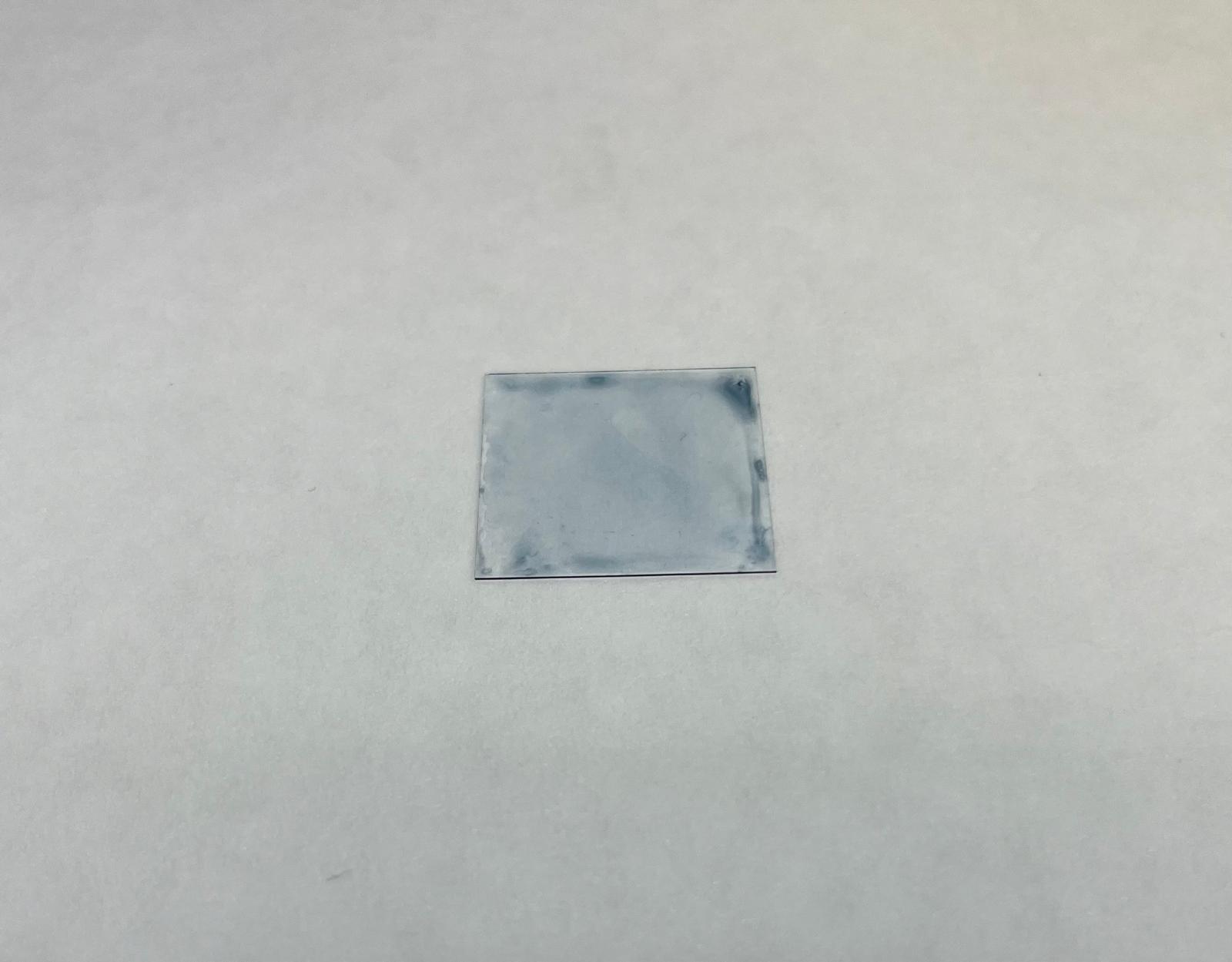

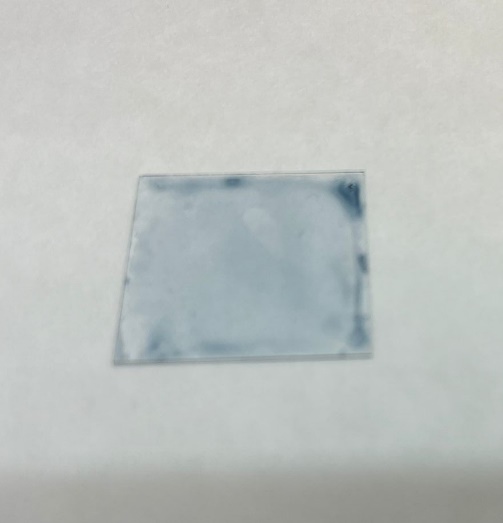


**Figure S6**. **Heat-treated in-house synthesized PEDOT:PSS pre and post wash**. **a**, Heat-treated in-house synthesized PEDOT:PSS spin-coated film before wash. **b**, Heat-treated in-house synthesized PEDOT:PSS spin coated film after wash.


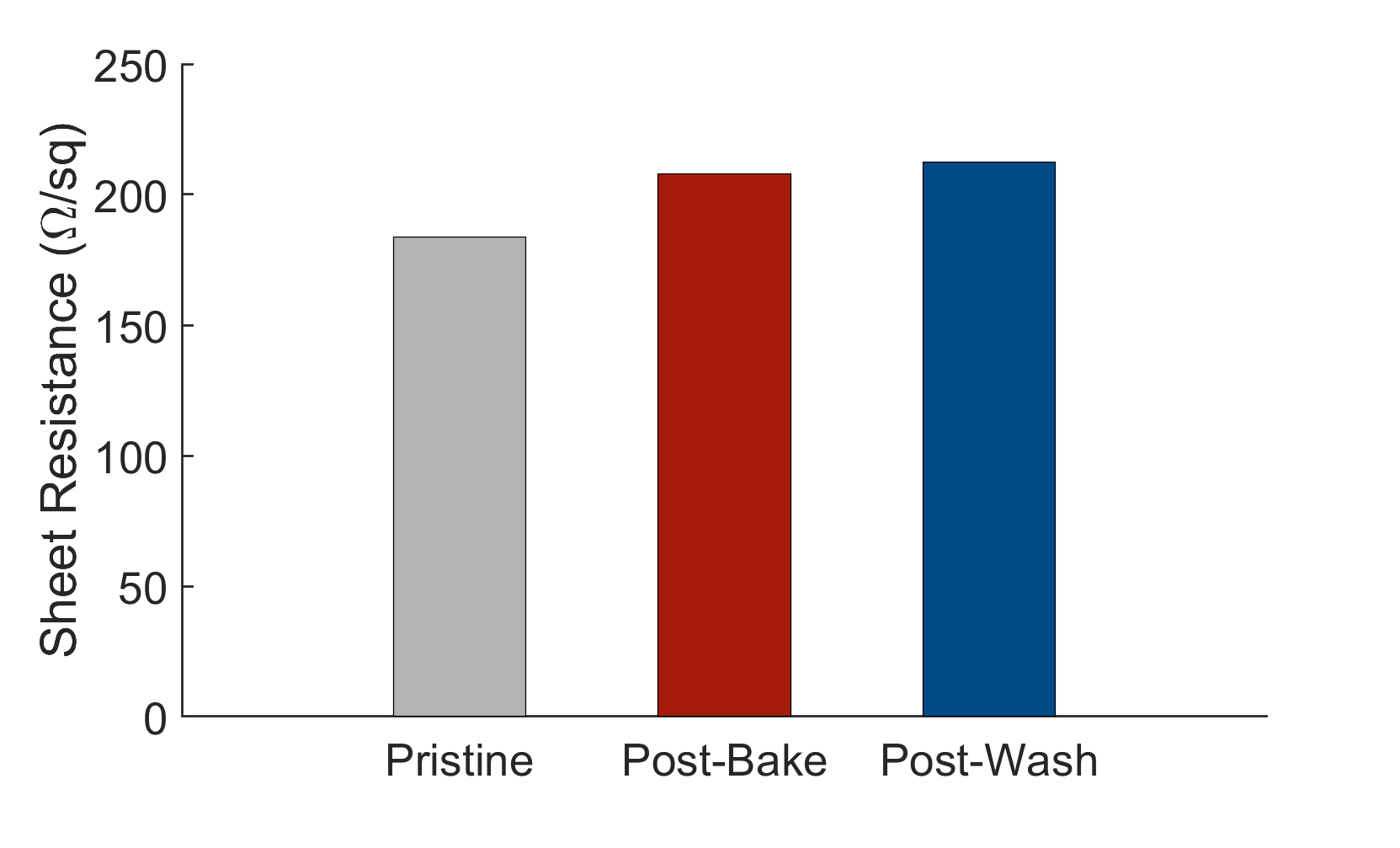


**Figure S7. Sheet resistance of heat treated PEDOT:PSS films with conductivity enhancing additive**. Films were prepared on glass from a PEDOT:PSS dispersion with 6% v/v ethylene glycol (EG), and then baked at 150 °C for 2 minutes prior to 4-pt probe measurements of sheet resistance.


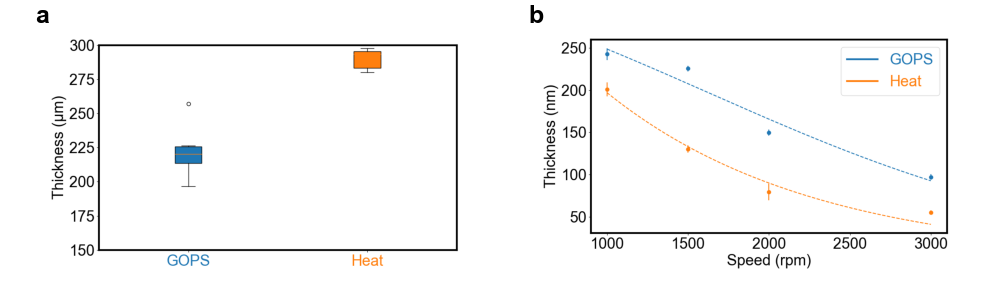


**Figure S8. Comparison of heat-treated and GOPS-crosslinked thicknesses with different spin coating parameters. a**, Resulting thickness (nm) of PEDOT:PSS for 1500 RPM, 5000 RPM s^-1^ acceleration, 5 s spin for heat-treated and GOPS-crosslinked PEDOT:PSS. **b**, Resulting thickness (nm) of PEDOT:PSS for 1000 RPM s^-1^ acceleration, 5 s spin for heat-treated and GOPS-crosslinked PEDOT:PSS as a function of the spin speed (RPM).


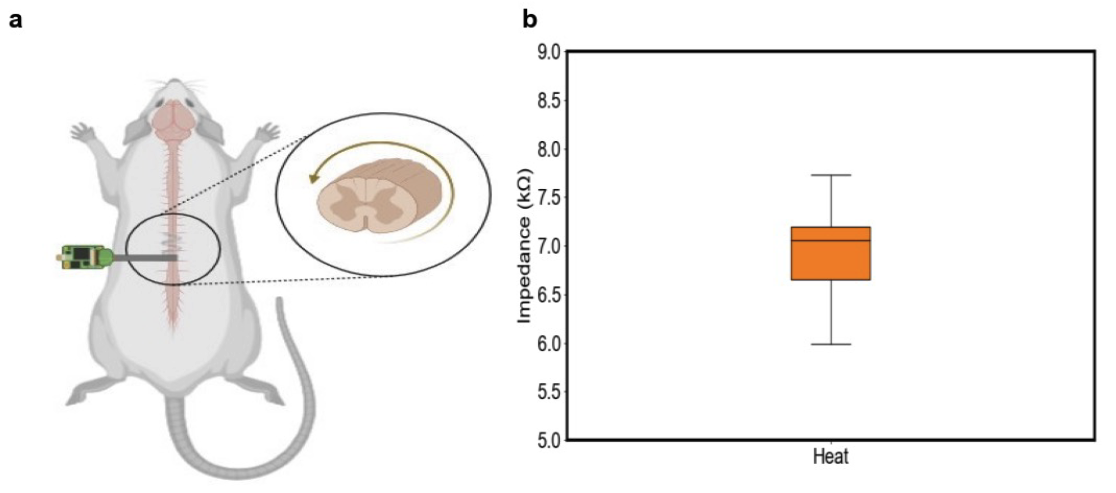


**Figure S9. Circumferential** **spinal cord stimulator implanted in an awaken rat. a**, Implantation schematic of the circumferential spinal cord stimulator and **b**, impedance (kΩ) distribution at 1 kHz of 10 channels picked at random after implantation around the spinal cord of an awaken rat.


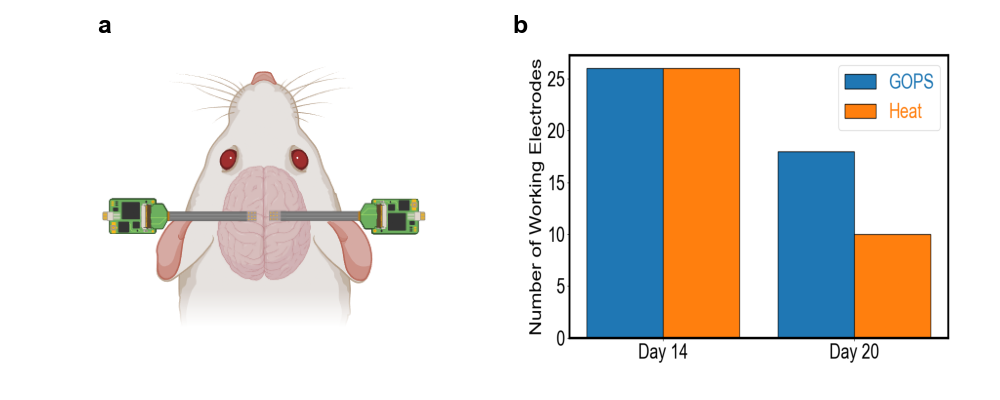


**Figure S10. ECOG device implantation schematic and working electrodes count. a**, Implantation schematic of the ECOG devices **b**, Number of working electrodes (<500 kΩ) at day 14 and day 20 for GOPS-crosslinked and heat-treated devices.


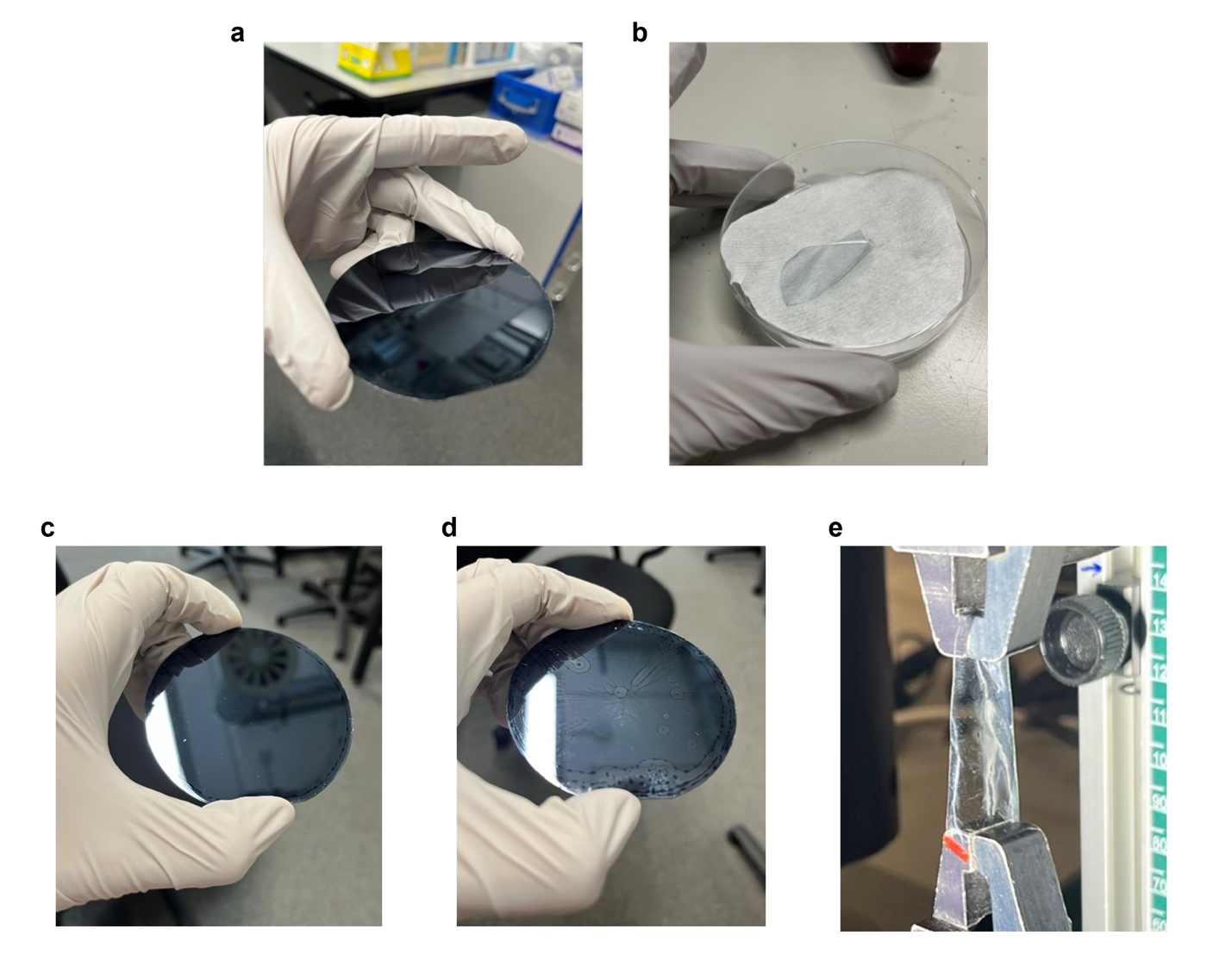


**Figure S11. Heat treated PEDOT:PSS on SEBS and PDMS substrates. a,** SEBS 25% in toluene film coated with heat-treated PEDOT:PSS after wash. **b,** SEBS 25% in toluene peeled off film with heat-treated PEDOT:PSS after wash. **c,** SYLGARD 184 film coated with heat-treated PEDOT:PSS after wash, **d**, SYLGARD 184 film with GOPS-crosslinked PEDOT:PSS after wash. **e,** SYLGARD 184 peeled off film with heat-treated PEDOT:PSS after wash.


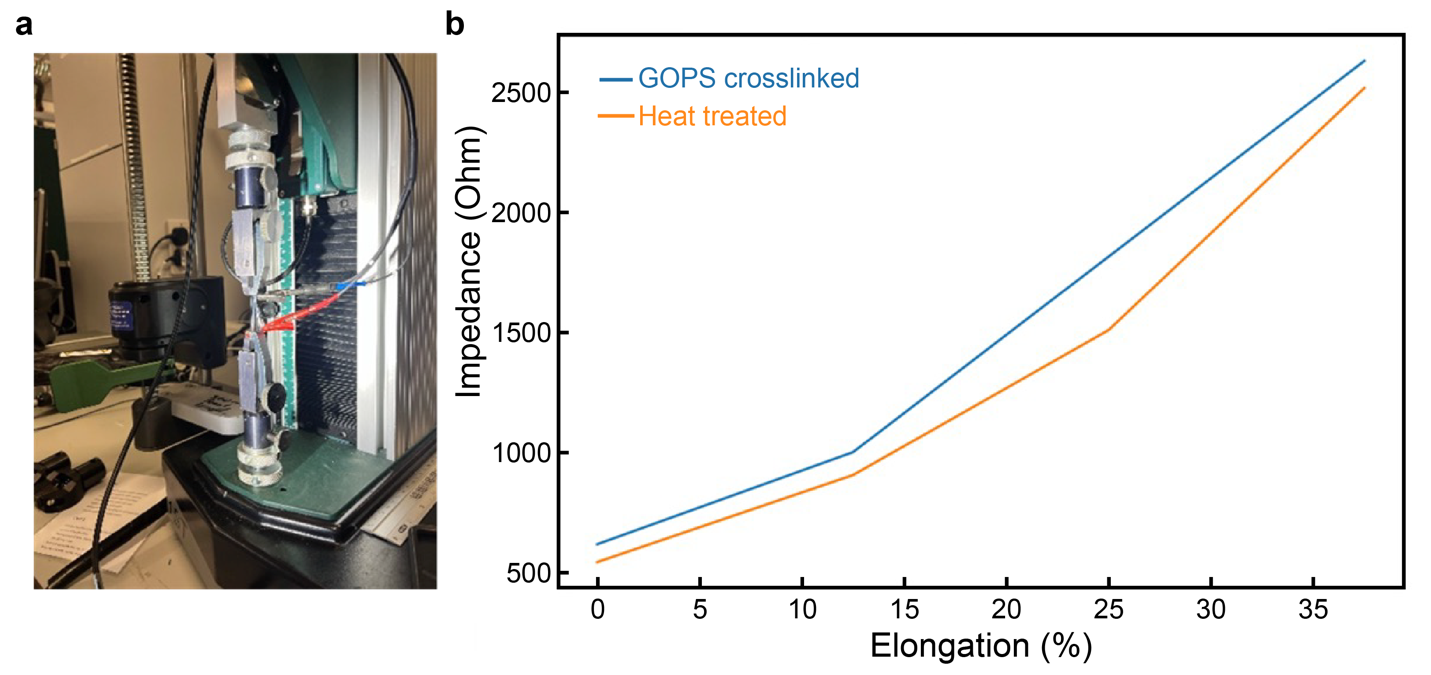


**Figure S12. Stretching of PEDOT:PSS on PDMS elastomeric substrates. a**, Setup for the stretching test. **b**, Elongation against impedance at 1kHz for a 2 cm wide, 4 cm long thin film of PEDOT:PSS on PDMS.


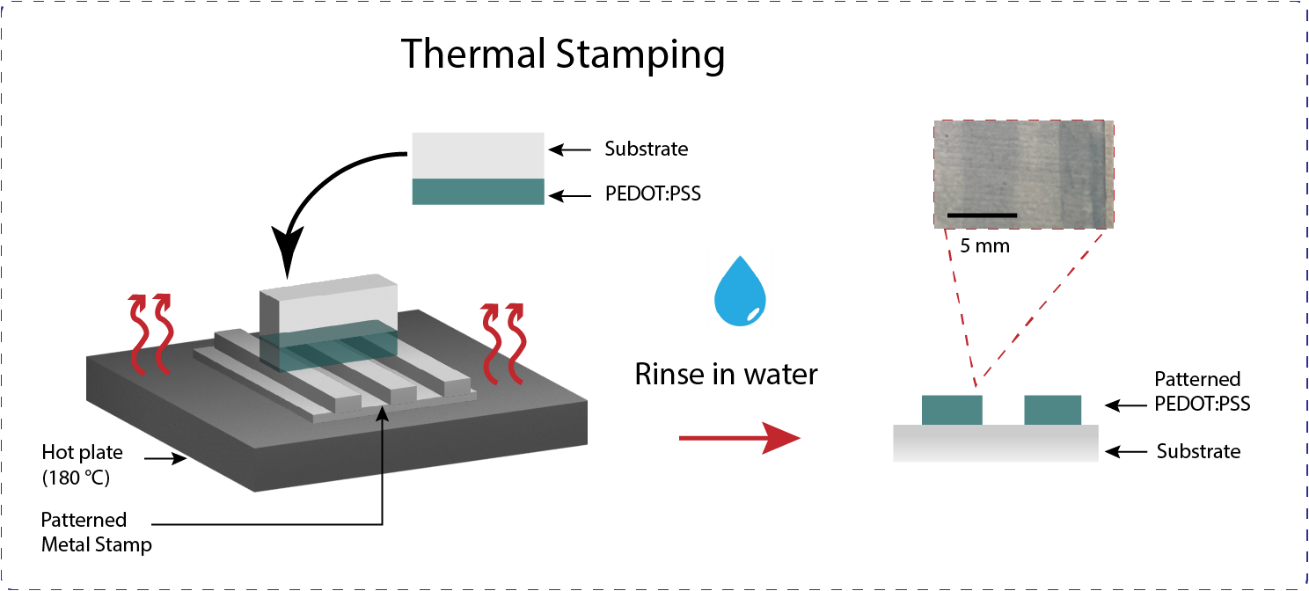


**Figure S13**. **Proof of concept for thermal-stamp based patterning**. Schematic demonstrating the localized application of heat using a patterned metal stamp. Inset demonstrates a mm- scale PEDOT:PSS line pattern written using this approach. The stamp was set on a hotplate heated to 180 °C and left to equilibrate for 10 minutes. Then the PEDOT:PSS film was annealed on the heated stamp for 5 minutes.


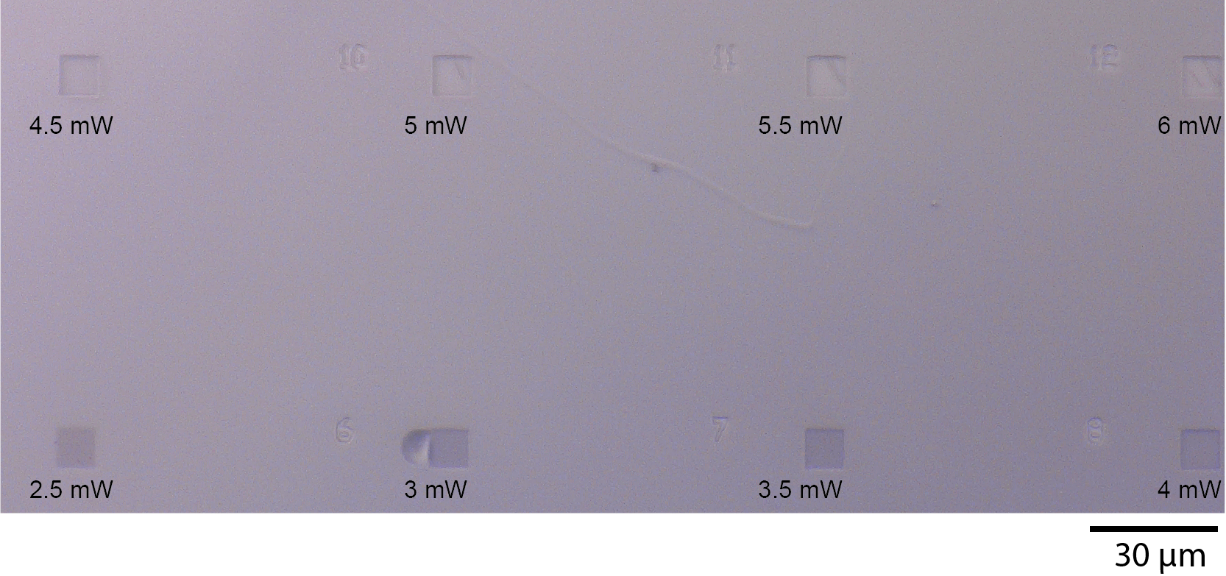


**Figure S14.** **Dose response for femtosecond laser writing of PEDOT:PSS**. An optical micrograph of 10 µm by 10 µm squares written at different laser intensities at a scan speed of 100,000 mm s^-1^ using a 63$\times$ objective.


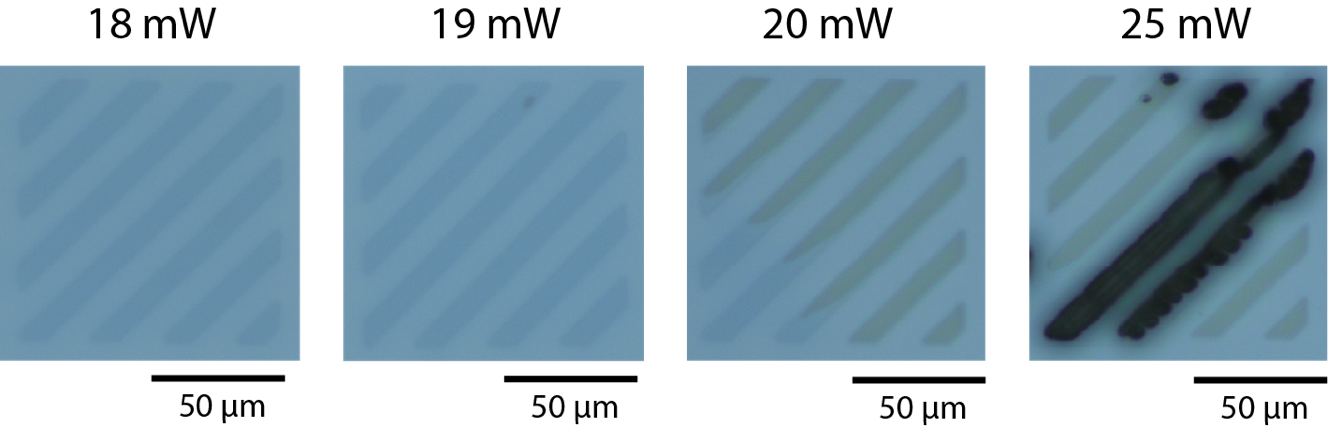


**Figure S15.** **Femtosecond laser writing of PEDOT:PSS on PDMS elastomer**. An optical micrograph of grating structures with 10 µm linewidths and 20 µm periodicity written at different laser intensities at a scan speed of 5,000 mm s^-1^ using a 20$\times$ objective. The PDMS elastomer substrate was prepared by spin coating PDMS onto an Si wafer.


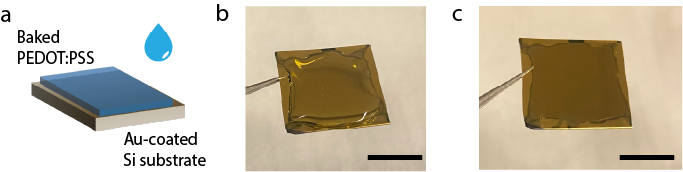


**Figure S16. Water stable PEDOT:PSS films on Au**. **a**, PEDOT:PSS films were prepared on an Au-coated Si substrate (3 nm Ti, 100 nm Au deposited by electron-beam evaporation) and heated for 2 minutes at 180 °C. **b**, Exposure of film to water. The film was immersed in a beaker with water for 90 s prior to the image being taken. **c**, film remains stable after water exposure. The scale bar for **b,** and **c,** is 1 cm.


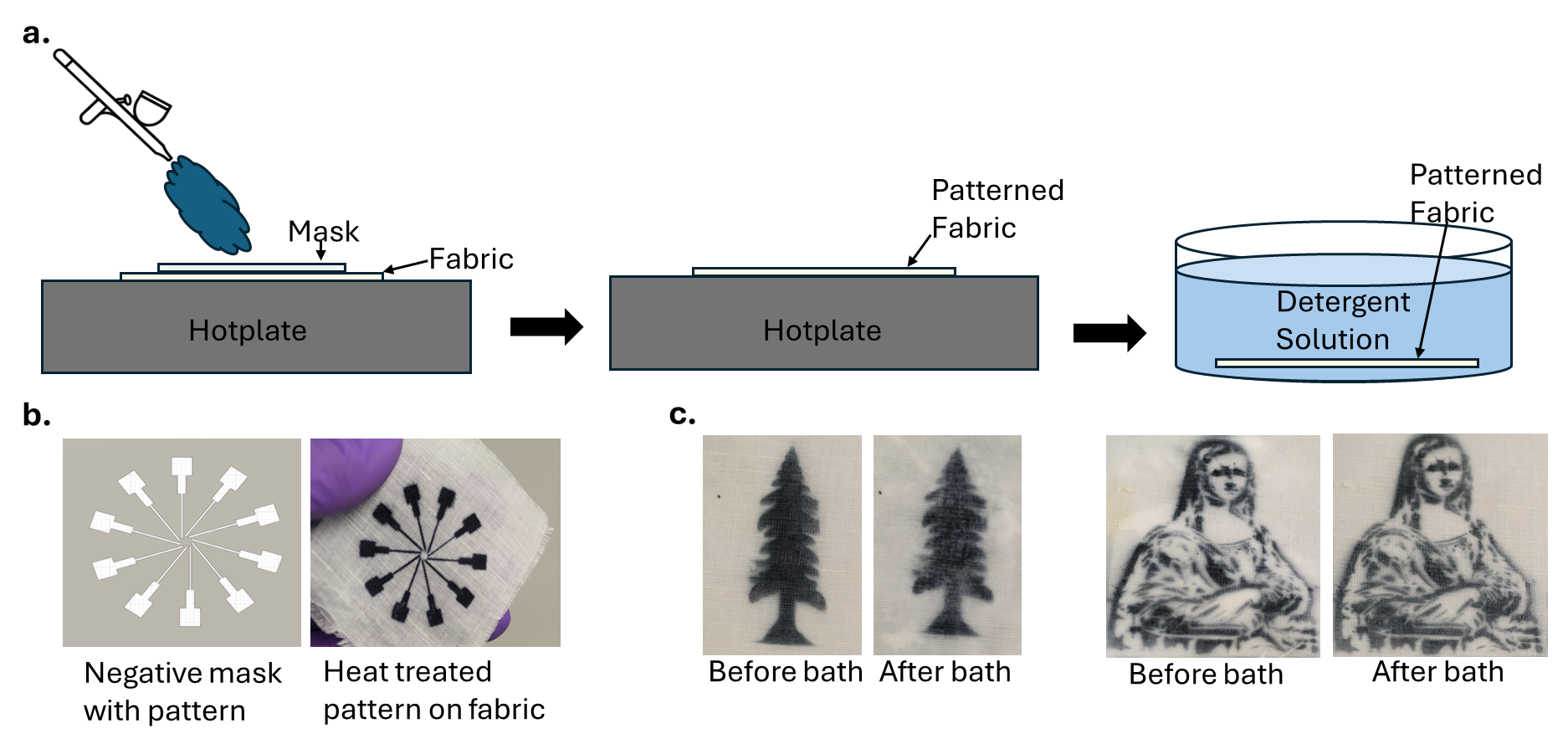

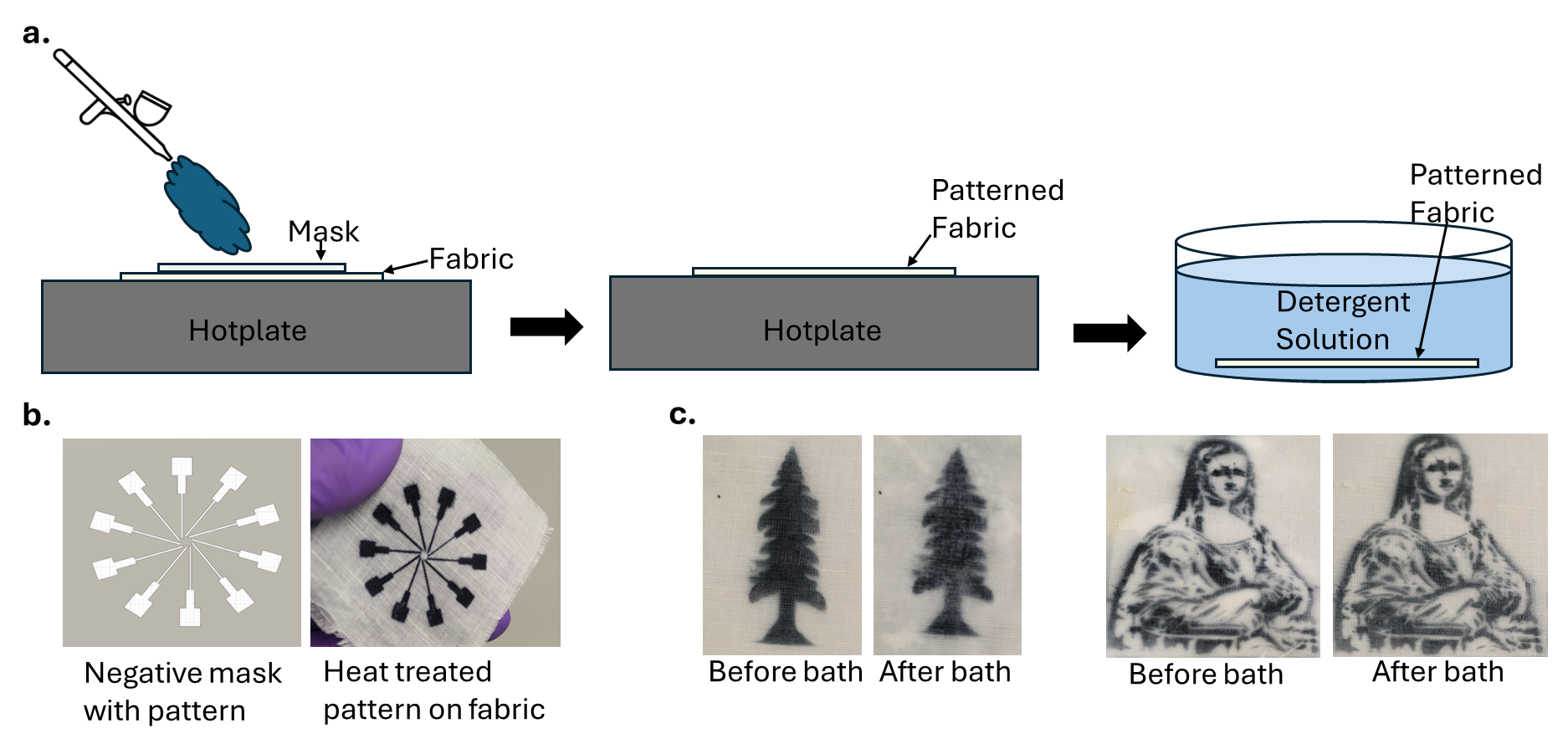


**Figure S17**. **Water and detergent stable PEDOT:PSS patterns on fabric**. **a**. PEDOT:PSS patterning on fabric (linen) with an airbrush and negative mask. The patterned PEDOT:PSS is heat-treated at 180 °C for 15 minutes. The patterned fabric is placed in a detergent solution on a shaker plate for 40 minutes at 100 rpm to test the stability of the pattern. **b**. Example of a negative mask and a heat-treated pattern on fabric. **c**. Example of patterned PEDOT:PSS on fabric before and after detergent bath.


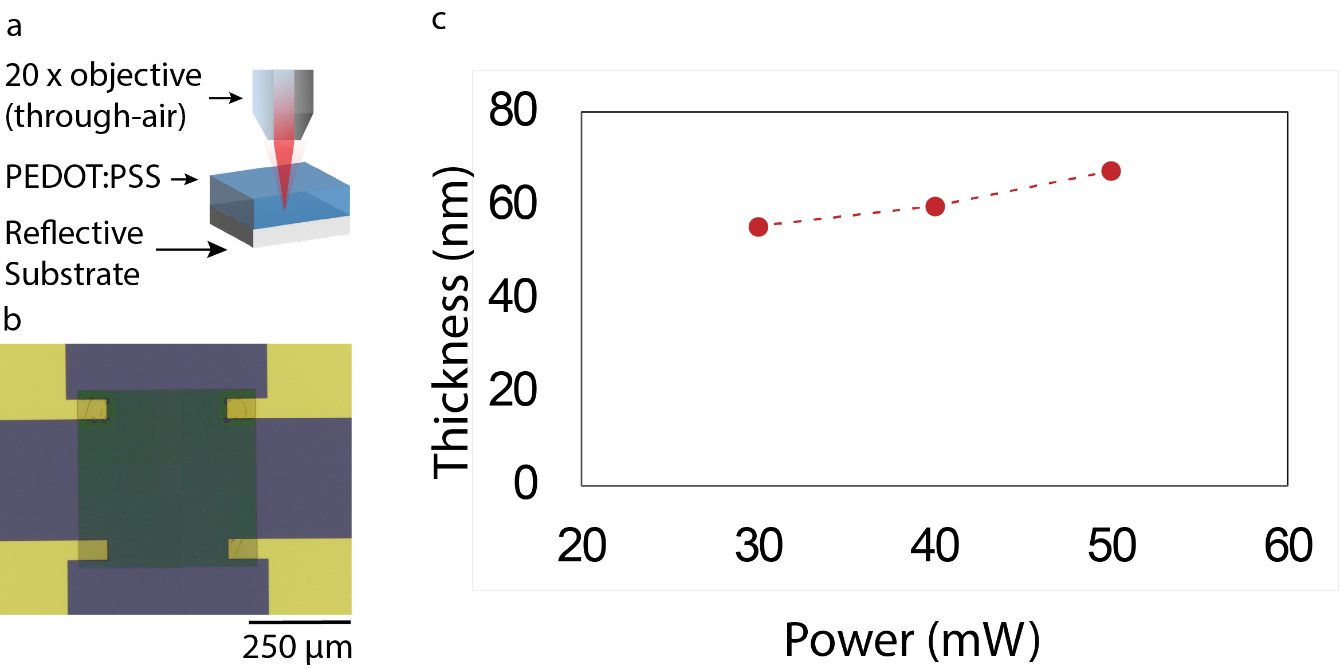


**Figure S18.** **Femtosecond laser** **written PEDOT:PSS on reflective, insulating substrates with lithographically patterned gold contact pads for van der Pauw measurements. a,** Square samples of 400 µm side length were prepared on a substrate consisting of thermally grown SiO_2_ on an Si wafer using an in-air 20 x objective at a scan speed of 5,000 mm/s. **b,** An optical micrograph of a sample prepared with 40 mW applied power. **c,** AFM measurements of the post-washing thickness of femtosecond laser written samples prepared at different powers. The samples were then used for conductivity measurements in **Fig. 4d**.


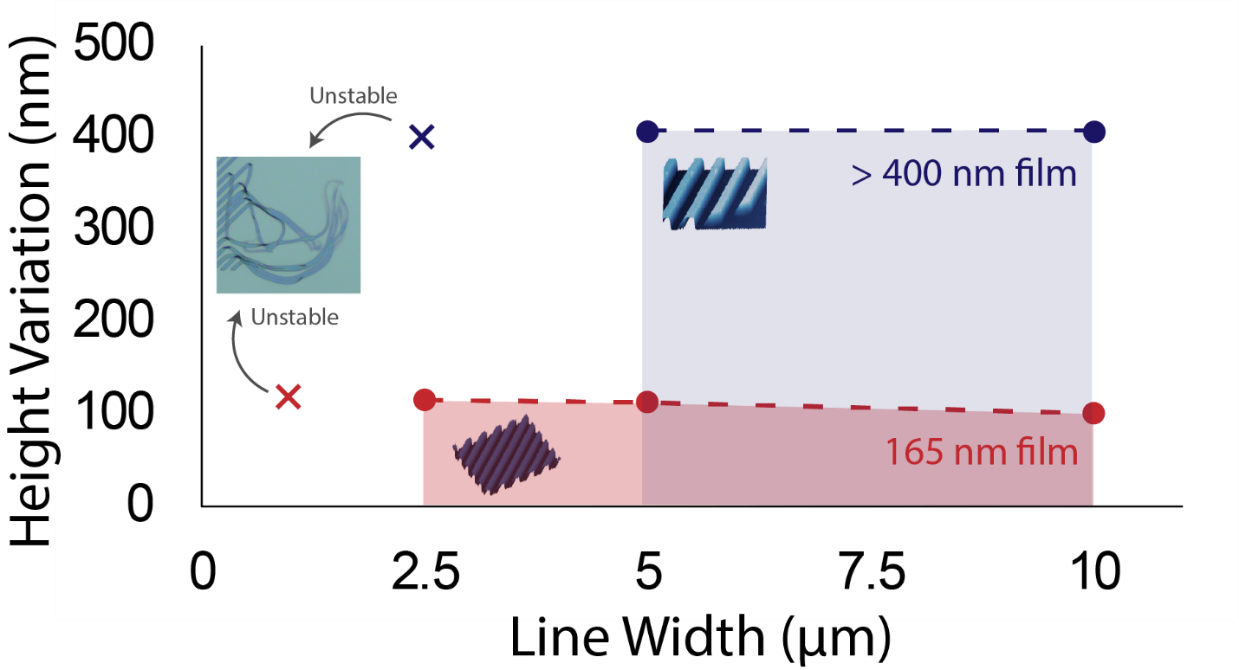


**Figure S19.** **Femtosecond laser writing of periodic nano-wire structures from thick PEDOT:PSS films**. Films spin-coated from viscous, high-solid content dispersions of PEDOT:PSS, prepared by rotary evaporation of commercial PH1000 dispersions, were patterned with a femtosecond laser. The as-cast thicknesses of the films were 165 nm in one case (red) and > 400 nm in the second case (blue). Thickness variations across the sample prevented precise determination of thickness for the patterned region for the >400 nm film. The eventual pattern thickness is lower than the thickness of the initial film, likely due to loss of PSS during washing. A modulation transfer function for the writing process, illustrating the variation of achievable contrast with feature size is plotted, where the linewidth is 50 % of the pattern periodicity. AFM images providing examples of the written structures are included as insets to the lower right of the data point corresponding to their write condition. The points plotted as crosses represent conditions where the pattern could not be accurately reproduced due to the formation of spaghetti-like structures after washing in water (example optical micrograph included as inset).


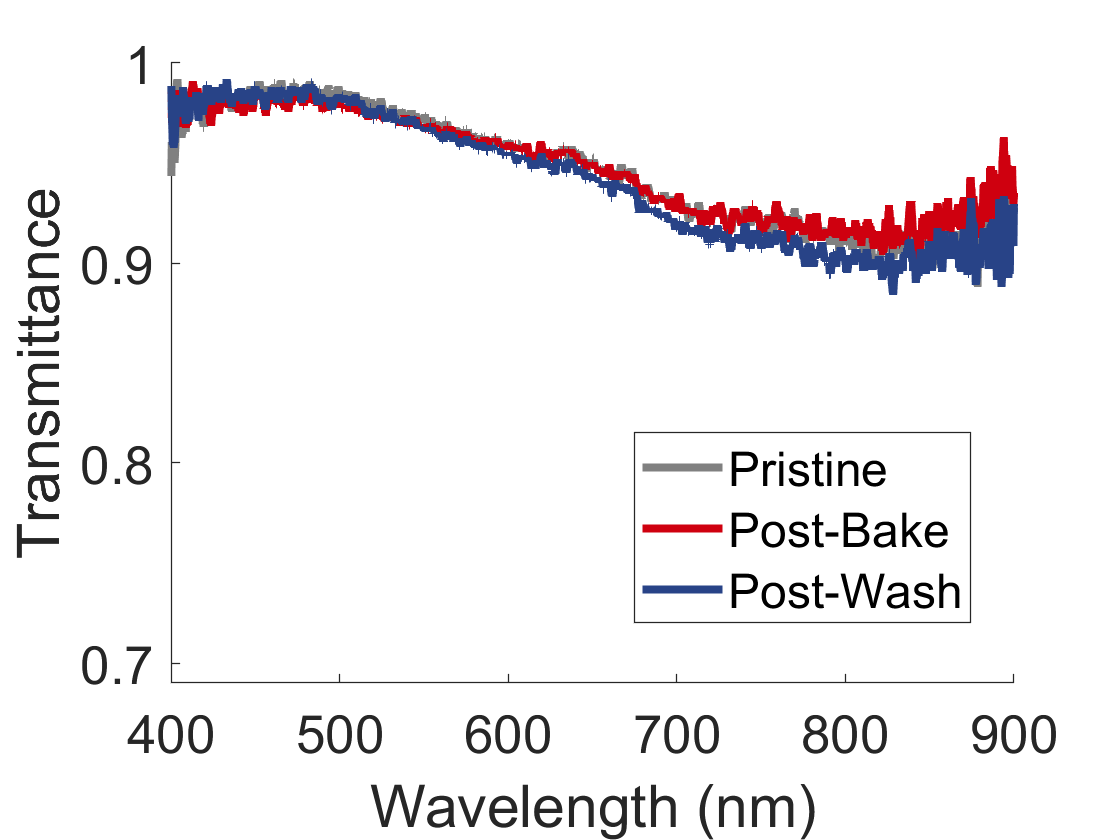


**Figure S20**. **UV-Visible spectroscopy of PEDOT:PSS films**. Effect of baking for 2 minutes at 180 °C on the transmittance of *ca.* 90 nm thick PEDOT:PSS films, both prior to and after exposure to water. The transmittance of the PEDOT:PSS films on glass were referenced to the transmittance measured through the bare glass substrate.

References

[1] M. Kong, M. Garriga, J. S. Reparaz, M. I. Alonso, *ACS Omega* **2022**, *7*, 39429.

[2] S.-Y. Lien, P.-C. Lin, W.-R. Chen, C.-H. Liu, K.-W. Lee, N.-F. Wang, C.-J. Huang, *Crystals* **2022**, *12*, 1109.
